# Evaluation of the diagnostic reliability of an AI prototype in detecting clinical features from dental photographs—original research

**DOI:** 10.3389/fdmed.2026.1814876

**Published:** 2026-05-08

**Authors:** Saif Abdelaziz, Islam Yasser, Mazen Amgad, Mutaz Al Qudah, Rohit Kunnath Menon

**Affiliations:** College of Dentistry, Ajman University, Ajman, United Arab Emirates

**Keywords:** artificial intelligence, deep learning, dental photography, diagnostic reliability, machine learning

## Abstract

**Purpose:**

This study aimed to evaluate the diagnostic reliability of a prototype artificial intelligence (AI)-assisted software for automated detection of clinical features from dental photographs, using sensitivity, specificity, positive predictive value (PPV), and negative predictive value (NPV) as metrics. Targeted features included caries, gingival recession, calculus, retained roots, bleeding, and staining.

**Methods:**

A cross-sectional study was conducted on 34 patients at Ajman University's College of Dentistry, generating 306 standardized intraoral photographs. Ground truth was established by clinical examiners and compared against the prototype software output. Diagnostic performance was analyzed using Stata version 15.0 (StataCorp).

**Results:**

The prototype demonstrated variable diagnostic performance. The prototype demonstrated higher sensitivity in detection of bleeding (71.43%; 95% CI: 68.74–74.11) and retained roots (72.73%; 95% CI: 66.48–78.98) with the lowest values reported for the detection of staining (24.38%; 95% CI: 21.63–27.13). The prototype demonstrated high specificity for detection of staining (98.77%; 95% CI: 98.11–99.42) and bleeding (98.88%; 95% CI: 98.15–99.45). The highest PPV was reported for the detection of staining (94.78%; 95% CI: 93.45–96.10), whereas bleeding (99.81%; 95% CI: 99.56–100.00) and retained roots (99.72%; 95% CI: 99.48–100.00) reported NPVs above 99%.

**Conclusion:**

The AI software demonstrates strong specificity and NPV, indicating reliable disease exclusion. Lower sensitivity and PPV suggest the need for algorithmic refinement to enhance detection performance and reduce false positives.

## Introduction

In dentistry, artificial intelligence (AI) assisted tools are transforming patient care by improving patient care, treatment planning, and diagnostic accuracy (1, 2). Real-time decision assistance, disease pattern recognition, and dental data analysis facilitated by machine learning and deep learning models have revolutionized dental applications. AI has shown the ability to enhance clinical judgment and optimize processes (3, 4), lowering the need for manual interpretation (1, 2, 5) in fields including orthodontics (4), periodontics (4, 6, 7), and restorative dentistry (8–10). AI has been widely used in medical imaging in the healthcare industry for tasks pertaining to organ segmentation, radiographic interpretation, and tumor identification (11–15). Large amounts of imaging data can be quickly and accurately analyzed by AI systems, which may also spot subtle disease signs that human observers may fail to identify (16–19). Despite this promise, research has revealed that many commercial AI models perform worse than expert evaluations when externally validated (11, 12, 15), underscoring the significance of thorough clinical validation and a diverse dataset.

AI applications are increasingly applied in dental radiography and intraoral scanning to identify anatomical abnormalities (4, 5), periodontal bone loss, and caries (20–30). Cone beam computed tomography (CBCT) scans, bitewings, and panoramic radiographs are analyzed to aid in enhancing diagnostic accuracy for medical professionals (12, 13). Recent systematic evaluations have alluded to the increasing efficacy of AI-assisted clinical intraoral photography for the identification of dental diseases. Preliminary findings from tele dentistry research suggest that photographic methods may attain clinically acceptable diagnostic efficacy, considering significant methodological variability and issues over study quality are progressively overcome (31). AI-centric evaluations have demonstrated significant discrepancies in research design, absence of standardization, and inferior methodological rigor (25, 32). AI-assisted caries detection has been proposed to be feasible and clinically pertinent, even though inherent constraints of inconsistent reporting, inadequate external validation, and deficiency of substantial real-world datasets remain as obstacles (33). Intraoral photography is a practical, non-invasive method with growing relevance in telehealth environments, although it remains limited by inconsistencies in acquisition protocols and reference standards (34). Analysis of intraoral clinical photographs are increasingly being employed in teleconsultation platforms and remote dental care with real benefit to end users (35–40). Standardized or patient-taken photos are used by other proprietary AI systems to evaluate the state of soft and hard tissues (41–43). Previous studies have identified shortcomings in certain parameters related to detection of crowding and gingivitis, frequently because of poor generalization, overfitting, or insufficient training datasets, even though some algorithms report excellent sensitivity and specificity (44, 45). Visual complexity and unpredictability in clinical photography as compared to radiographic analysis further enhance the challenges pertaining to the reliability of the diagnostic findings. There is a discernible lack of validated research especially addressing the diagnostic reliability of AI systems employing clinical pictures in standard dentistry settings, despite the widespread use of AI technologies in dental diagnostics (46–50). Although spectrophotometers are the benchmark for objective colour measurement, their application in clinical diagnostics is constrained. Smartphone and Digital Single-Lens Reflex (DSLR) photography facilitates comprehensive dental evaluation, with no consistent advantage of one technology over the other. The integration of artificial intelligence, rather than the imaging technology, is the principal factor influencing diagnostic performance (49, 51, 52).

Many of the studies that are now available either use carefully selected datasets, only consider radiographic data, or evaluate a small number of clinical factors. Furthermore, the computational architecture and training data of commercial products are frequently opaque. Independent, clinically based assessments that compare AI technologies to gold-standard examiner evaluations under real-world circumstances are not available.

The purpose of this study was to evaluate the diagnostic reliability of a prototype AI system, in detecting a range of common dental clinical features—including caries, recession, calculus, retained roots, staining, and bleeding—from standardized intraoral photographs. By comparing AI-generated outcomes with expert examiner assessments, this research provides insight into the strengths and limitations of clinical image-based AI diagnostics.

## Materials and methods

A cross-sectional study design was employed to evaluate the reliability of the prototype software. Ethical approval for the study was obtained from the Research Ethics Committee, Ajman University (D-F-H-24-10-31-a). The study population comprised patients aged 18 years and older presenting at the College of Dentistry, Ajman University. The sample size was determined according to the anticipated prevalence and precision outlined in a prior research by Bonfanti-Gris et al. (44). To enhance the robustness of the findings and account for the simultaneous occurrence of the relevant diseases in a single picture, the study used 300 photos. Criteria for inclusion: (1) Patients presenting to the College of Dentistry at Ajman University for routine treatment and who furnish written informed permission. (2) Individuals aged 18 years and older. Criteria for exclusion: (1) Patients necessitating urgent medical intervention. (2) Patients who have received scaling and root planing during the past 3 months. All patients received a comprehensive explanation from the research team regarding the research objectives and their participation; the confidentiality of their photos was assured. Informed consent was secured from each subject prior to picture acquisition following the explanation.

The photographs were captured with a Sony Alpha a7 IV mirrorless digital camera, equipped with a Sony FE 90 mm F2.8 Macro G OSS lens and a flash. Godox MF12 Dual Macro Flash (The flash intensity settings employed were: 1/2 for anterior and lateral images; and 1/1 for upper and lower occlusal photographs) Shutter speed: 1/125 s, Aperture: f/22, ISO: 160. One operator captured all photographs, and the same mirrors and image collection techniques were utilized for each patient to guarantee uniformity. Intraoral occlusal photos of the maxillary and mandibular arches were captured and categorized into three sections: anterior, right posterior, and left posterior. A total of 34 patients were featured, resulting in 306 photographs.

Each photograph was reviewed by two reviewers (IY, SA) to confirm the existence of a minimum of three identification criteria as detailed below in a single photograph: Caries: C; Gingival recession: R; Pit and fissure caries/stain: P; Calculus: K; Retained roots: X; Smooth surface stain: S; Bleeding: B; Missing tooth: M. The indices selected to assess the clinical conditions had to rely on visible features to be applicable for photographic diagnosis. International Caries Detection and Assessment System (ICDAS) was the agreed reference standard for caries assessment (53). Millers’ classification to assess recession (54). Simplified Calculus Index (CI-S) to assess calculus deposits (55). Two independent examiners (IY, SA) evaluated all photographs and documented the findings according to established criteria. Each finding was discussed and a consensus was achieved before documentation. Any discrepancies or disagreements were reviewed and confirmed by the third examiner (RKM) following the same assessment criteria. If a consensus was not achieved concerning any photograph, that photograph was excluded. The diagnostic consensus was defined as the “ground truth.” Thereafter, the photos were uploaded to the prototype software system. The photograph's quality remained unaltered to maintain consistency between the evaluator's identification environment and the prototype. The images uploaded to the prototype gadget were anonymized and promptly erased once the research concluded to safeguard participants’ privacy and confidentiality.

**Figure 1:**
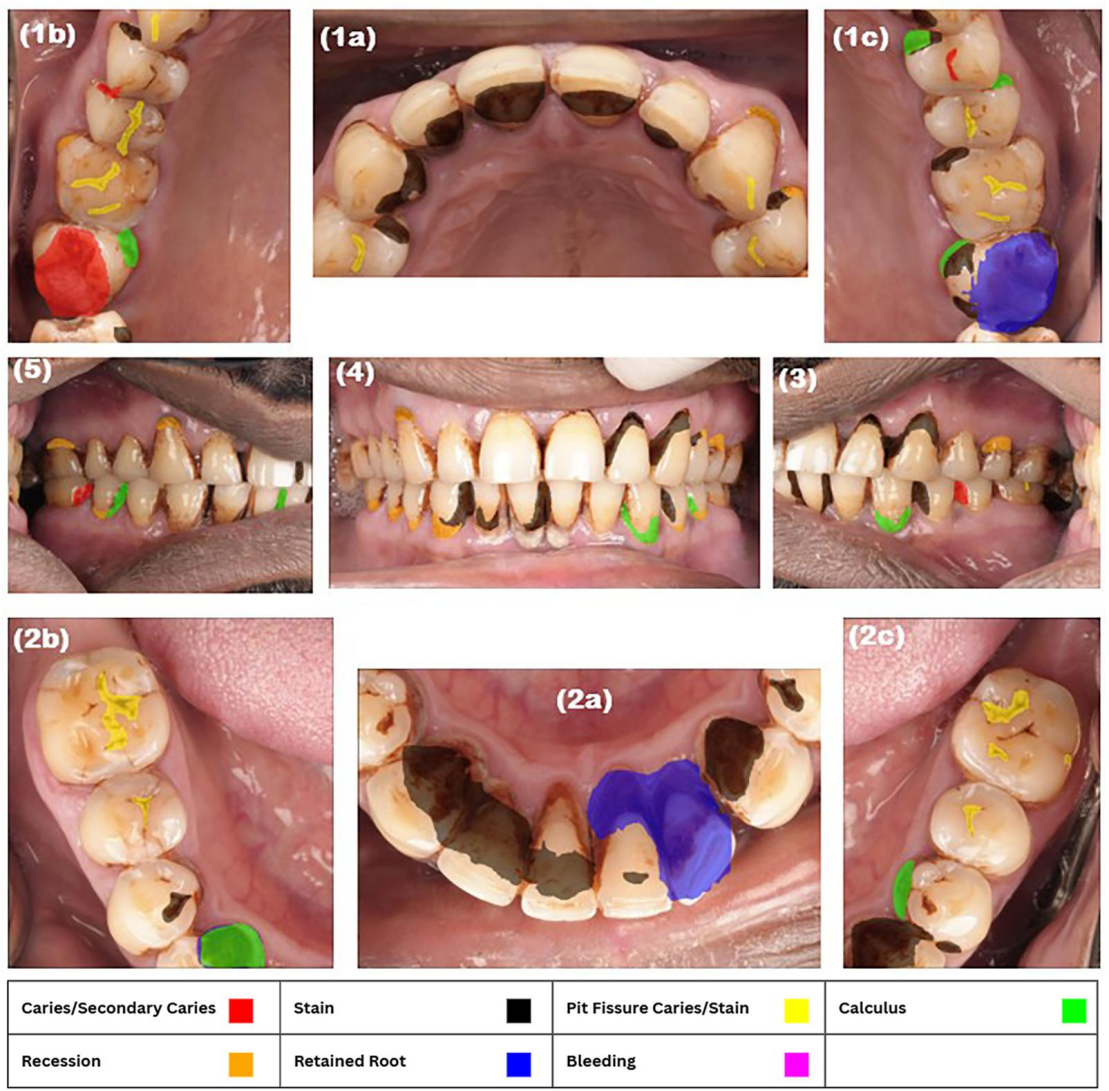
Examples of photos with AI detection. **(1a)** maxillary anterior teeth, **(1b)** maxillary right posterior teeth, **(1c)** maxillary left posterior teeth, **(2a)** mandibular anterior teeth, **(2b)** mandibular right posterior teeth, **(2c)** mandibular left posterior teeth, **(3)** lateral left view, **(4)** anterior view, **(5)** lateral right view.

A sample image of the AI software systems output can be observed in (Figure 1).

The performance of the automatic detection stage was evaluated on the basis of detection sensitivity, which was calculated as the number of teeth/factors automatically detected by the software out of the number of teeth/factors identified as ground truth. The diagnostic sensitivity (S), specificity (E), positive predictive value (PPV), and negative predictive value (NPV) of the tested dataset was calculated (44, 45) by using using Stata version 15.0 (StataCorp) for statistical analysis and graph generation for every study variable. A descriptive analysis was performed as well.

## Results

Table 1 presents the values for the test characteristics across all outcomes. The prototype's diagnostic performance exhibited variability across outcomes, with sensitivity values spanning from 24.38% (CI: 21.82–26.93) for staining to 72.73% (CI: 70.08–75.37) for retained roots, with bleeding showing a relatively high sensitivity of 71.43% (CI: 68.74–74.11). Throughout all outcomes, specificity consistently exceeded 92% for most parameters, varying from 73.71% (CI: 71.09–76.32) for recession to 98.80% (CI: 98.15–99.45) for bleeding and 98.77% (CI: 98.11–99.42) for staining. The negative predictive values were consistently elevated across all clinical parameters, surpassing 90% for the majority of outcomes and attaining 99.81% (CI: 99.56–100.00) for bleeding and 99.72% (CI: 99.40–100.00) for retained roots, demonstrating robust reliability in ruling out illness. Conversely, positive predictive values exhibited significant heterogeneity, spanning from 17.00% (CI: 14.77–19.23) for pit and fissure caries/stain to 94.78% (CI: 93.45–96.10) for staining. A subsequent subgroup analysis was conducted following the exclusion of undetected missing teeth by the prototype. Supplementary Table S2, illustrating the findings of the subgroup analysis, can be found in the Supplementary Material. ROC curves and AUC values for all results are reported in the Supplementary Material.

**Table 1:** Overall results for diagnostic performance of the model.

Outcome	Sensitivity %	Specificity %	PPV %	NPV %	Prevalence %
B (O)	71.43 (68.74, 74.11)	98.80 (98.15, 99.45)	27.78 (25.12, 30.44)	99.81 (99.56, 100.00)	0.64 (0.17, 1.12)
C (O)	33.03 (30.23, 35.82)	95.71 (94.51, 96.91)	46.15 (43.19, 49.12)	92.77 (91.23, 94.31)	10.02 (8.23, 11.80)
K (O)	35.91 (33.06, 38.76)	92.52 (90.96, 94.08)	60.00 (57.09, 62.91)	82.21 (79.94, 84.48)	23.81 (21.27, 26.34)
P (O)	46.58 (43.61, 49.54)	83.65 (81.45, 85.84)	17.00 (14.77, 19.23)	95.61 (94.39, 96.83)	6.71 (5.22, 8.20)
R (O)	60.62 (57.72, 63.53)	73.71 (71.09, 76.32)	28.45 (25.76, 31.13)	91.57 (89.92, 93.22)	14.71 (12.60, 16.81)
S (O)	24.38 (21.82, 26.93)	98.77 (98.11, 99.42)	94.78 (93.45, 96.10)	58.78 (55.77, 61.63)	47.89 (44.92, 50.85)
X (O)	72.73 (70.08, 75.37)	97.59 (96.67, 98.50)	23.53 (21.01, 26.05)	99.72 (99.40, 100.00)	1.01 (0.42, 1.61)

B, bleeding; C, caries/secondary caries; K, calculus; P, pit and fissure caries/stain; R, gingival recession; S, staining; X, retained root; O, overall.

## Discussion

The study aimed to assess the diagnostic reliability of a prototype software system in the automated recognition of clinical features from dental photographs using artificial intelligence. The authors performed an independent validation and were not involved in the development of the prototype.

**Figure 2:**
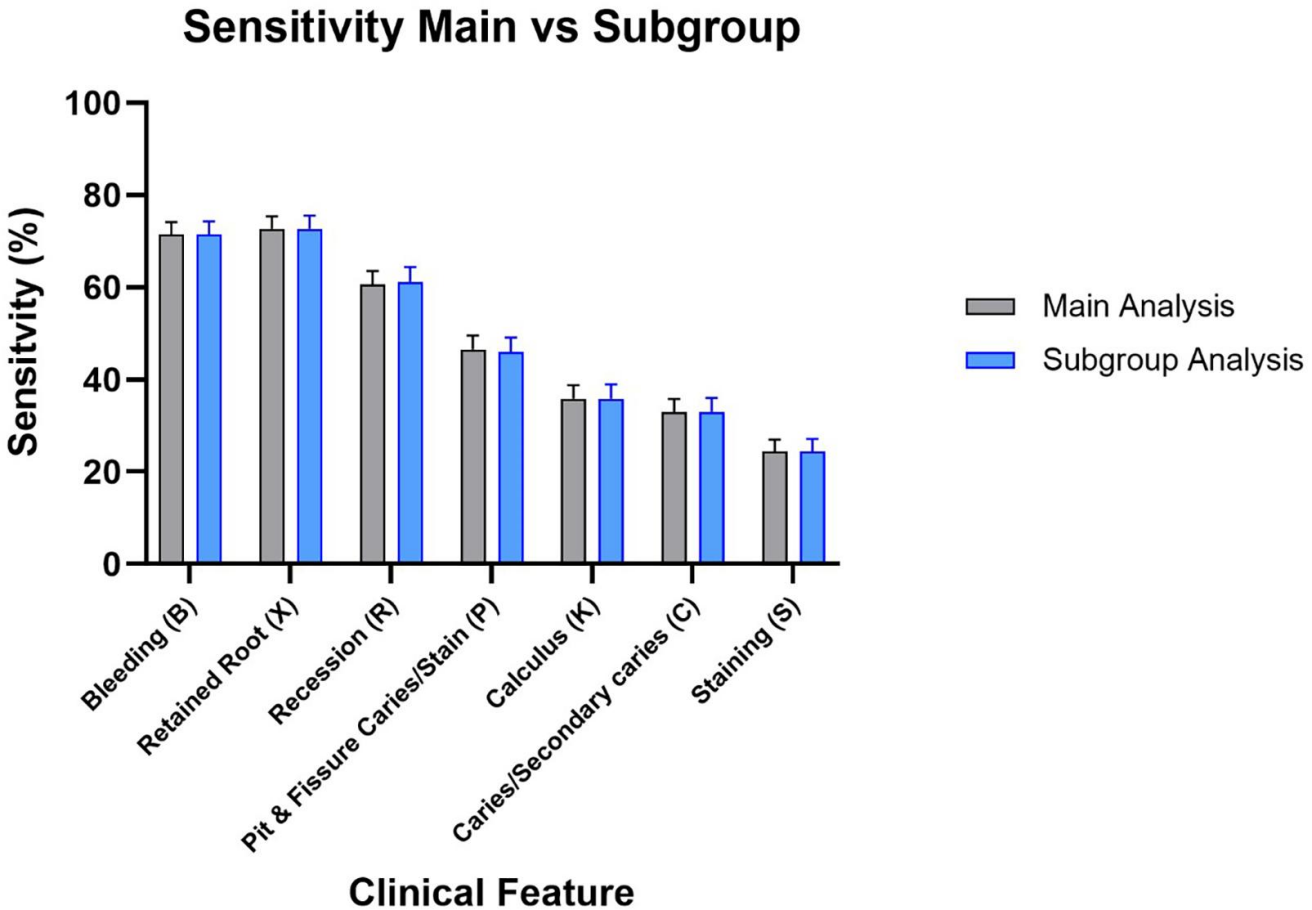
Sensitivity results for main group and subgroup analysis.

**Figure 3:**
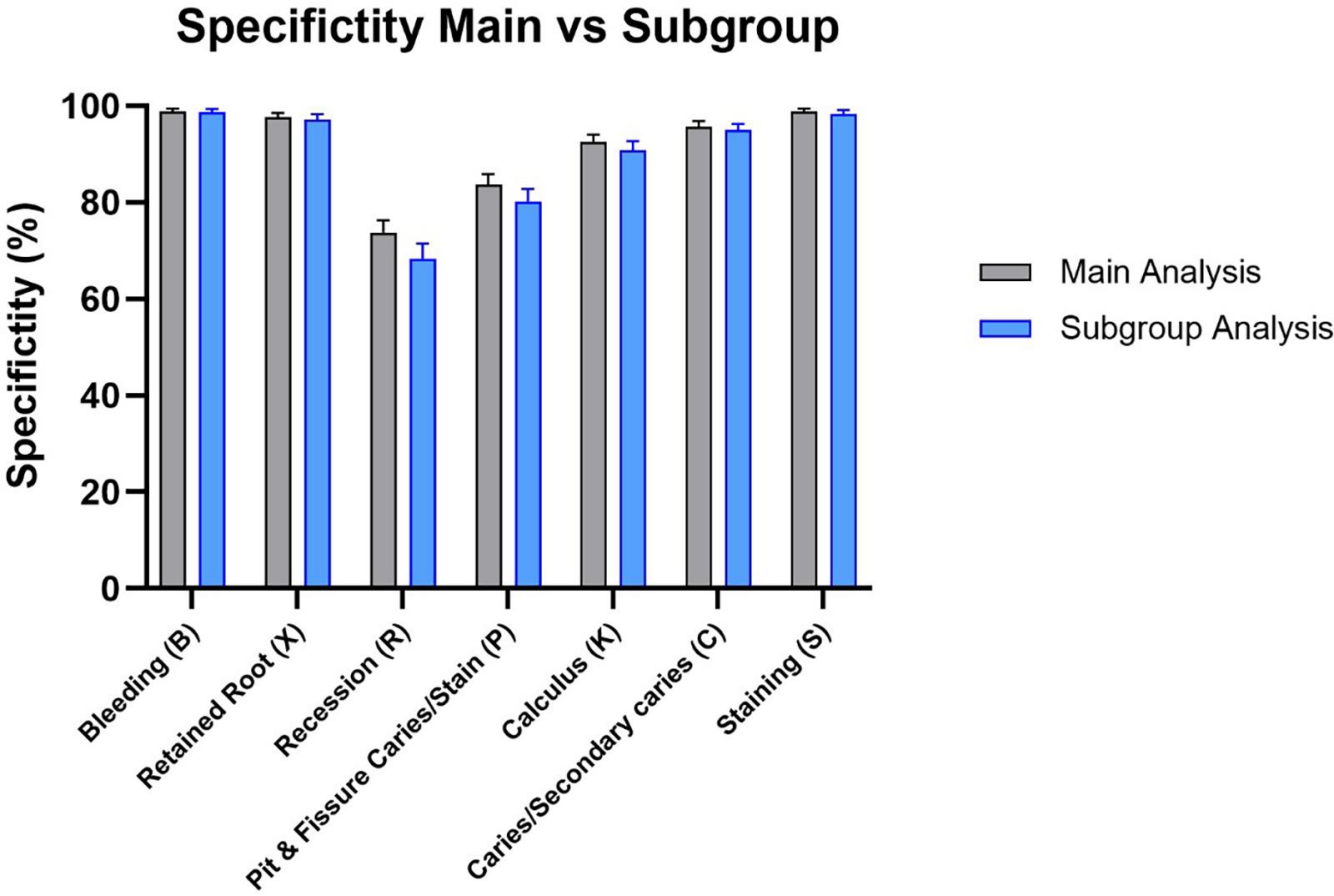
Specificity results for main group and subgroup analysis.

**Figure 4:**
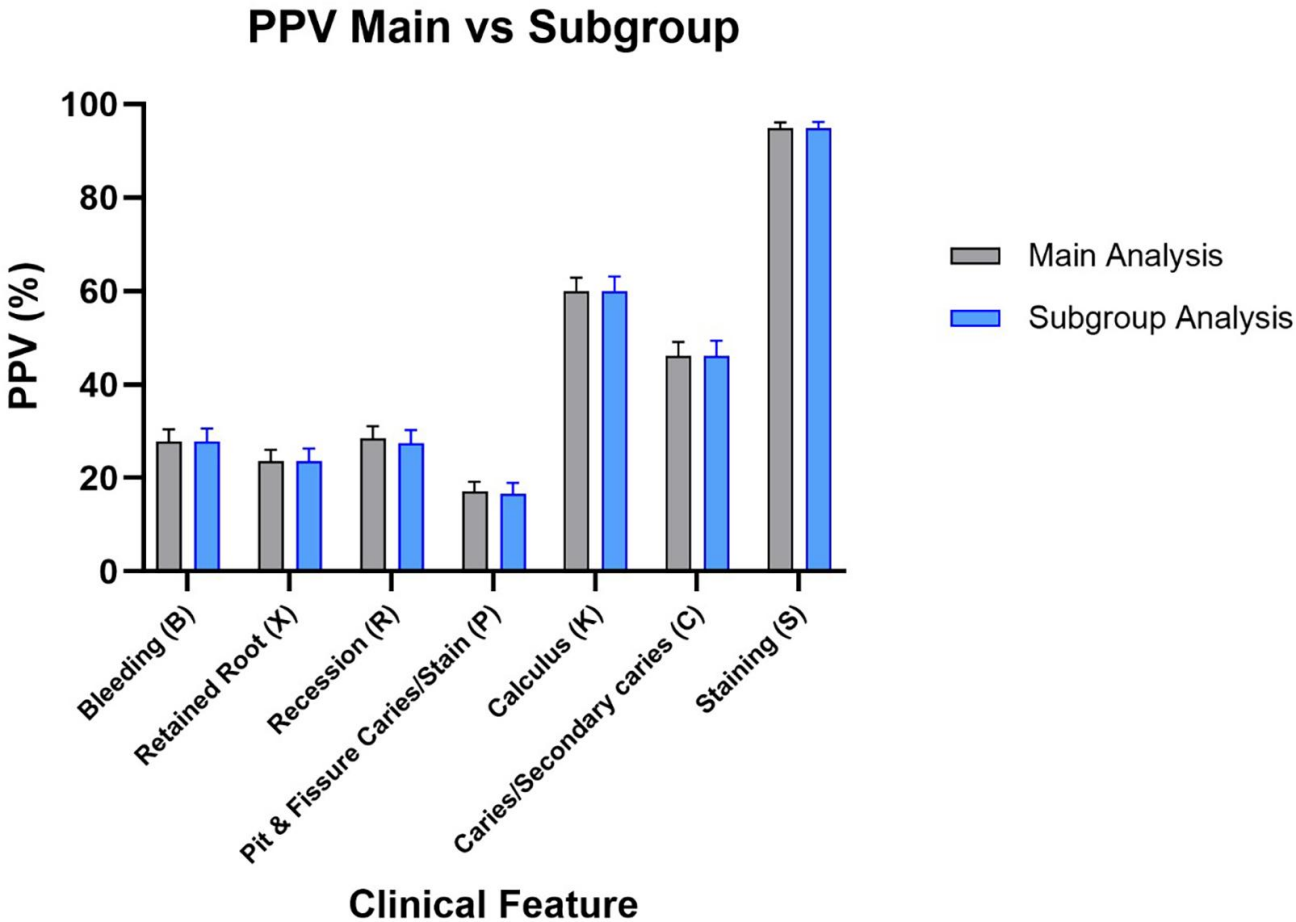
PPV results for main group and subgroup analysis.

**Figure 5:**
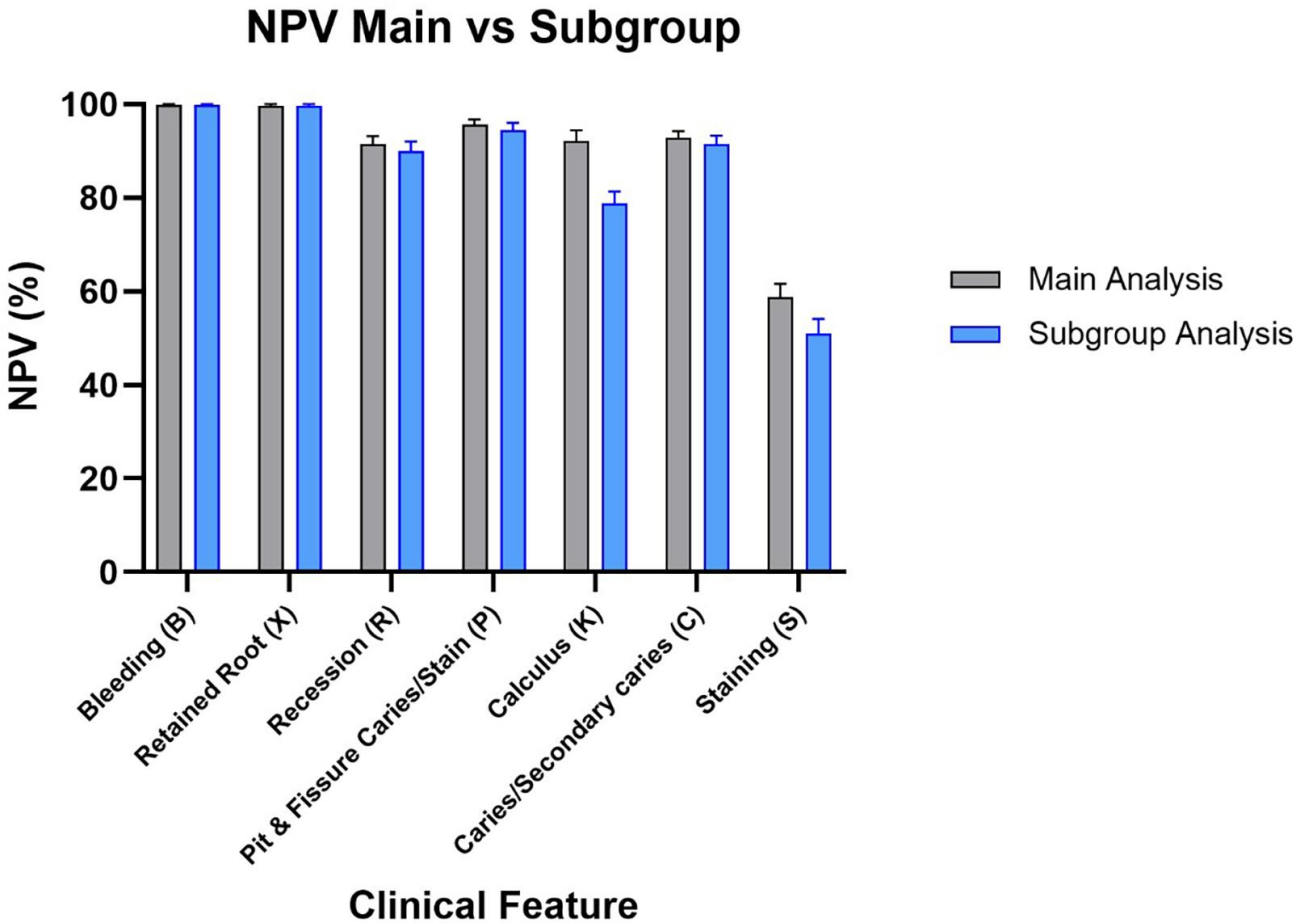
NPV results for main group and subgroup analysis.

The diagnostic assessment of the prototype software demonstrates ability to rule out disease, as indicated by the consistently elevated negative predictive values (NPV) across most test parameters. The elevated NPV values indicate that the program is dependable in identifying persons who do not display the tested characteristics. In the primary analysis, missing teeth were included in the denominator for several tooth-dependent variables. The subgroup analysis, excluding the missing teeth, validated the overall patterns observed in the primary analysis, visualized in Figures 2–5.

The regional analysis indicates that the diagnostic effectiveness of the prototype varied according to the anatomical location within the oral cavity. The mandibular posterior segments typically exhibit greater sensitivity than the anterior segments. This trend was evident for pit and fissure caries, stains, and calculus. These findings correspond with clinical predictions, as posterior teeth display deeper fissures, broader occlusal surfaces, and a more pronounced plaque-retentive structure, hence increasing the visibility of cues to the algorithm (46, 49). The maxillary anterior segments demonstrated increased specificity in identifying staining, bleeding, and retained roots, likely owing to improved image quality and more consistent lighting during the photographic acquisition procedure. Improved tooth morphology and reduced shadows may augment the model's edge detection and color contrast recognition. The detection of recession demonstrated limited sensitivity across all regions, with no specific segment exhibiting a markedly improved performance. However, specificity decreased in the anterior regions, particularly in mandibular anterior teeth, which may be attributed to the perceived similarity between healthy physiological gingival contours and actual pathological recession (42). The regional results demonstrate that the diagnostic efficacy of the prototypes is influenced by the clinical attributes or existing disease, in addition to the anatomical intricacy and photographic availability of the pertinent area. This underscores the need for region-specific training data and suggests that uniform performance cannot be expected when assessing the complete dental arch (44). Specific regions occasionally demonstrate the lack of pertinent clinical features implying low prevalence, even when 300 photos were utilized in accordance with the recommended sample size calculation. Clinical signs like calculus, stains, or cavities are region-specific. Sites are controlled by stable biomechanical and ecological constraints like plaque stagnation, morphology, saliva flow/clearance, gingival phenotype, and accessibility for self-care. The oral cavity contains certain “high risk” sites; including occlusal pits and fissures for caries, lingual mandibular anterior and buccal maxillary posterior surfaces for supragingival calculus (56, 57), and buccal (often mid-buccal) sites for gingival recession (58).

A recent systematic review evaluated the accuracy of AI models in detecting periodontal disease using digital intraoral images (34). The model (Dental Monitoring, Paris, France) performed similarly to the current investigation by focusing on gingivitis and periodontitis without additional classification (34).

The investigation revealed that clinical features had a lower occurrence compared to healthy surfaces, affecting the positive predictive value (PPV) and retaining a high negative predictive value (NPV), often exceeding 90%. This indicates a dependable model for reporting disease absence, attributed to high specificity and low disease prevalence. In low-prevalence scenarios, even tests with moderate sensitivity yield significant NPVs, making them effective for screening (43–45). Conversely, PPV was generally poor, correlating with disease rarity and resulting in more false positives. Staining demonstrated a higher PPV despite poor sensitivity, underscoring this relationship.

The ROC curves presented in the Supplemental Materials demonstrate the model's ability to discriminate between diseased and non-diseased instances for conditions like bleeding, retained roots, and recession, showing significant distinctions with curves shifting to the upper left. In contrast, characteristics associated with caries, such as smooth surface and pit and fissure caries, showed ROC curves closer to the diagonal line, indicating limited ability to differentiate subtle carious lesions. The current findings align with literature noting reduced diagnostic accuracy in photographic caries detection (24, 25, 27, 30–33, 36, 38, 39, 43, 49). The ROC analysis suggests that adjusting thresholds could improve sensitivity for certain conditions (48, 49), highlighting the potential for enhanced model efficacy by calibrating thresholds based on clinical context, such as prioritizing sensitivity in public health vs. specificity in chairside testing. AUC values showed varying discriminatory performance across all diagnostic parameters. Bleeding (AUC = 85.11) and retained roots (AUC = 85.16) demonstrated good discrimination, whereas caries, calculus, and staining demonstrated moderate performance (AUC range: 61.57–67.17). The comparatively lower AUC values show inadequate overall diagnostic discrimination, especially for mild or early-stage diseases, despite continuously good specificity across the majority of outcomes. There appears to be a systematic model bias toward ruling out illness rather than identifying real positive instances, evident by the consistently smaller and different confidence intervals for sensitivity compared to specificity. The overall trustworthiness of these results is supported by the small confidence intervals across most measures, which show steady estimates.

Qualitative examination reveals a consistent correlation between the AI-generated picture overlays and the output identified by the prototype. The model exhibited robust performance in recognizing diseases linked to specific color alterations, contour disruption, or localized structural loss, including staining, bleeding, and retained roots. The model consistently emphasized these traits across several perspectives in different parts of the mouth. In contrast, errors occurred more frequently in situations necessitating nuanced interpretation of surface texture or small variations. Caries and pit and fissure lesions were occasionally misclassified or totally overlooked. This is particularly evident when discoloration is mild or influenced by lighting conditions or reflections. Significantly, false positives were predominantly concentrated in regions affected by shadowing. The model may be overly responsive to variations in contrast rather than authentic illness characteristics. This qualitative discovery corresponds with the low positive predictive value noted for various situations and offers a significant pathway for the future enhancement of the models’ algorithms. A training set with greater diversity in photographic circumstances may be beneficial.

The prototype performs better in excluding conditions (high NPV and specificity), although it needs additional optimization to augment sensitivity and boost PPV for various test parameters. This will enhance overall diagnostic precision and work to eliminate false positives and false negatives, hence facilitating its practical implementation in dental diagnostics. The software exhibited great specificity under all settings, demonstrating robust performance in accurately identifying healthy individuals. However, sensitivity was significantly low across various dimensions, indicating the software does not identify certain actual positive situations.

Low sensitivity raises the possibility of underdiagnosis, especially in tele-dentistry or community-based settings where these devices can be utilized for initial screening. In a screening context, this carries direct patient safety implications, low sensitivity leads to missed diagnoses (false negatives) This may result in patients being falsely reassured that they are disease-free, reducing the likelihood of seeking further clinical evaluation. Delayed intervention is a major consequence. Undetected conditions may progress to more advanced stages before being identified clinically, often requiring more invasive, costly, and complex treatments. In dentistry, this could mean progression from early enamel caries to dentinal involvement or pulpal pathology. Low sensitivity can also contribute to inappropriate clinical decision-making, especially if clinicians over-rely on AI outputs. If the system incorrectly indicates absence of disease, it may influence clinicians, particularly less experienced practitioners, to overlook subtle clinical signs. This may also introduce ethical and medico-legal implications. Missed diagnoses attributable to AI systems may raise concerns regarding accountability, informed consent, and standard of care, especially if the limitations of the system are not clearly communicated. Low sensitivity undermines the effectiveness of AI as a screening tool, as screening technologies are typically designed to prioritize sensitivity over specificity to minimize the risk of missed disease. As a result, it is not advised to use the present system as a stand-alone diagnostic tool, and clinical validation by qualified experts is still crucial. The clinical aspects that received lower scores necessitate additional assessment methods for accurate and reliable detection and diagnosis in a genuine clinical evaluation (43, 44).

Carious lesions in a clinical context would compel the physician to employ the International Caries Detection and Assessment System (ICDAS) alongside its International Caries Classification and Management System (ICCMS) (53). Dental caries is a prevalent condition that impacts humans and affects quality of life, necessitating the examination and comparison of results related to this condition. Despite using a complex architecture integrating Mask R-CNN (59) with a Swin Transformer backbone (60, 61), the prototype demonstrated lower caries detection sensitivity compared to CNN-based methods. Zhang et al. (27) tested a commercial artificial intelligence system (Aiyakankan, Aicreate, China) that used intraoral photographs to detect dental caries using a hybrid deep learning architecture that combined MobileNet-V3 for feature extraction with U-Net segmentation. Aiyakankan's model demonstrated a substantially greater sensitivity and PPV with similar specificity and NPV in detecting occlusal caries as compared to the current prototype. Jones et al. (29) employed an Attention UNet to categorize beginning, moderate, and advanced dental caries in children utilizing intraoral scans and 2D images. The model demonstrated similar strengths and limitations to the current prototype; however, direct comparison was not feasible as the author did not offer sufficient information on solely permanent dentition findings. The study by Estai et al. (43) employed smartphone cameras to capture images of teeth from 100 participants, the collection of photographs and their characteristics closely resemble those of this study. Dental examiners assessed caries presence from dental photographs, achieving sensitivity results in the ballpark of (60%–63%), which compared to the prototype model [46.58%, CI: (43.61, 49.54)] when detecting Pit & fissure caries/Stain and [33.03%, CI: (30.23, 35.82)] for Caries/Secondary Caries. Specificity results for photographic analysis by human dental examiners (96%–99%) and the prototype [Caries/Secondary caries 95.71%, CI: (94.51, 96.91)] and [Pit & fissure caries/Stain 83.65, CI: (81.45, 85.84)] are very similar. Current data indicates employing photos as a diagnostic medium for dental diseases, whether by seasoned practitioners or artificial intelligence, carries the danger of underdiagnosis (43). Moreover, a shared characteristic of these two studies and other pertinent research in the field is the capacity to exclude dental illness, which may possess intrinsic value (24, 25, 31, 32, 35, 36).

Sensitivity, specificity, and prevalence all influence positive predictive value (PPV) and negative predictive value (NPV). The positive predictive value increases with the prevalence of the outcome. The diminished positive predictive values for various outcomes in this study may result from the low prevalence of certain outcomes in relation to the total data points. Despite the test's efficacy in distinguishing between individuals with and without the condition, the quantity of true positives will significantly diminish relative to the amount of false positives in groups with low prevalence. Thus, despite high sensitivity and specificity, the positive predictive value diminishes (62).

The diagnostic consensus, or “ground truth,” for the present investigation was by three reviewers. The fundamental accuracy in radiographic identification from dental radiographs has been previously shown utilizing a comparable methodology (63, 64). The accuracy of every AI model is contingent upon the quality of the reference standard. The reference standard serves as the baseline for evaluating the index test, such as an AI model. In dental radiology, a definitive gold standard frequently does not exist. Consequently, professional interpretation serves as the practical benchmark (65, 66). Radiographic interpretation is intrinsically subjective, influenced by variations in reading experience, limits in picture quality, anatomical overlap, and the presence of small abnormalities. This results in inter-observer variability and inconsistent labelling (ground truth noise) (66). This underscores that each individual reader is inadequate as a dependable reference norm. A multi-reader method, defined as a label decided by the consensus of two or more readers, is favored since it facilitates adjudication, hence enhancing repeatability and agreement. To validate the reference standard, inter/intra-examiner reliability would serve as an effective instrument to minimize bias risk in the reference standard domain. Future additions of reviewers to the prototype may occur to guarantee consensus.

The prototype exhibited moderate sensitivity in diagnosing bleeding, recession, and retained roots. Patients typically perceive these symptoms without photographic analysis, and the motivation to pursue treatment may emerge naturally. Thus, the significance of precisely detecting such illnesses via automated methods remains contentious. A significant limitation and potential confounder may be the environmental lighting conditions during the photo capture; the specific lighting conditions of the room could have influenced the final photographic output and, consequently, the prototype's capacity to identify certain anomalies. This variable was deliberately left uncontrolled to replicate a realistic scenario in which this application would often be utilized, such as in domestic settings where the individual would use a mobile device for photography and self-assessment. In these circumstances, the lighting conditions would vary unpredictably among individuals and potentially from image to image; hence, we believe it is warranted to leave this variable unregulated to improve the study's generalizability. An expanded sample size obtained from diverse locales might enhance the generalizability of the data. Subsequent research ought to concentrate on exploring techniques to enhance the prototype's sensitivity. Another route to investigate is the extent to which patients recognize value in these diagnostic systems and how the intricacy of the diagnosed ailment affects this impression.

This study offers significant value by addressing various gaps through a clinically grounded assessment of an AI prototype utilizing standardized intraoral photographs and examiner-established ground truth across multiple prevalent dental conditions, including caries, calculus, gingival recession, retained roots, staining, and bleeding. This study assesses multi-condition diagnostic performance in real-world clinical settings, contrasting with previous research that primarily examines single conditions or controlled datasets. It presents thorough diagnostic accuracy metrics (sensitivity, specificity, PPV, NPV), thereby offering new insights into the practical reliability and limitations of AI-assisted diagnosis using clinical photographs.

The prototype utilized a Mask R-CNN structure with a Swin Transformer backbone (59, 67). This hybrid design integrates the advantages of region-based convolutional neural networks with transformer-based feature extraction, facilitating high-performance analysis of intraoral dental images. The system concurrently executes instance segmentation and object identification for accurate localization and delineation of problematic areas in dental imaging. This design was chosen due to numerous significant advantages. Mask R-CNN facilitates simultaneous detection and pixel-level segmentation, crucial for the precise identification of irregular and diffuse dental diseases. The Swin Transformer backbone improves feature representation by effectively capturing local texture details and global contextual linkages, surpassing traditional convolutional backbones (e.g., ResNet) in intricate visual domains like intraoral imaging. Additionally, the training pipeline is compatible with COCO-style evaluation measures, facilitating standardized performance assessment and adaptable optimization. The algorithm was trained on a meticulously selected collection of intraoral dental photos. The training dataset comprised of 1,255 photos, exhibiting a roughly balanced gender distribution (50.28% female and 49.72% male). The data was partitioned for model construction into training, validation, and test sets in an 80:10:10 ratio. Alongside condition-based classification, demographic diversity was maintained. The dataset encompassed variations in race, age, and gender; preserving this variety across all subsets mitigates bias and guarantees the model's effective generalization to diverse populations. A composite stratified sample method was employed to reconcile clinical and demographic factors. This amalgamated dental condition, together age and race, establishes stratification groups that provide equitable representation of uncommon conditions while preserving demographic equilibrium. Following the division, distributions were assessed to ensure that all pertinent parameters were adequately represented. A systematic and therapeutically directed annotation methodology was established to guarantee superior ground truth labelling. Annotators were licensed dentists possessing skill in oral diagnostics. The annotation type was Polygon-based instance segmentation masks (COCO format) (68, 69). Conventional systems facilitating the generation of polygon masks were employed as the annotation tools. All annotations were executed manually by dental specialists to ensure clinical validity. A multi-phase quality assurance procedure was utilized, incorporating cross-review and consensus validation to reduce inter-observer variability. The annotation schema comprised of Dental caries: Various manifestations and degrees of severity, Periodontal conditions: Gingival and periodontal anomalies, Observations: Plaque deposition and enamel staining. To guarantee uniform model input and optimal training efficacy, all pictures are subjected to a standardized preprocessing protocol before training and inference. Images were scaled to a predetermined resolution while maintaining aspect ratio. Pixel intensities were standardized to a uniform range. Images were padded to consistent dimensions for batch processing. Alongside preprocessing, data augmentation techniques were employed during training to improve robustness and mitigate overfitting. Colour jittering was utilized to introduce regulated alterations in brightness, contrast, saturation, and hue. This replicates variations in illumination conditions and imaging devices, hence enhancing the model's capacity to generalize across diverse clinical datasets. The amalgamation of sophisticated deep learning frameworks, clinically verified annotations, and a comprehensive preprocessing pipeline empowers the proposed dental AI system to provide precise and scalable identification of dental diseases in intraoral imaging. Numerous models were developed for distinct classes, each utilizing a portion of the full dataset. Although there was considerable overlap of pictures among many models, a rigorous distinction between training and validation data was upheld within each model to prevent data leaking. Initially, 600 photos were collected and classified into six criteria for clinical validation. Following the application of quality criteria, a subset of photos were selected and the distribution was ensured to illustrate both clinical attributes and data accessibility, with a greater number of samples for diseases such as caries and retained roots owing to their manifestation and relative occurrence.

The authors were not involved in the development of the protoype. Independent progressive validation of prototypes is essential to be reported and the data used for further improvement to maintain scientific transparency. Most research using AI-assisted diagnostic tools detect caries by approaches other than intraoral images. Limited research has emerged using artificial intelligence to identify periodontal disease. No research has evaluated further clinical features (recession, staining, retained root, etc.) in this manner. This study may represent initial efforts to evaluate supplementary clinical factors such as recession, staining, and retained root.

## Conclusion

The prototype exhibited good specificity and negative predictive value; however, its inadequate sensitivity for numerous critical situations constrains its independent clinical use.

The results indicate that the prototype is likely more appropriate as a supplementary screening instrument for excluding illness rather than for confirming diagnosis. Nonetheless, the potential for underdiagnosis continues to be a considerable issue, especially in extensive or community-oriented implementations. Future enhancements should concentrate on augmenting sensitivity via algorithm optimization and expanding the number and variety of training datasets to enhance model generalisability and detection efficacy.

## Data Availability

The original contributions presented in the study are included in the article/Supplementary Material, further inquiries can be directed to the corresponding author.
