# Supplementary material for: Evaluation of the diagnostic reliability of an AI prototype in detecting clinical features from dental photographs—original research
Source: Front Dent Med. 2026 May 8;7:1814876. doi: 10.3389/fdmed.2026.1814876 (PMC13194533; doi:10.3389/fdmed.2026.1814876)
Supplement: Supplementary file 1 [file supplementaryfile1.docx]

Supplementary Material

# Supplementary Data

# Supplementary Figures and Tables

Supplementary Table 1: Region-wise results for Main Analysis with Prevalence and AUC values.

| **Outcome** | **Sensitivity %** | **Specificity %** | **PPV %** | **NPV %** | **P**revalence % | **AUC Values** |
| --- | --- | --- | --- | --- | --- | --- |
| B (MP) | 66.67 (61.66,71.68) | 98.81 (97.67,99.96) | 33.33 (28.32,38.34) | 99.70 (99.12,100.00) | 0.88 (-0.11,1.88) | 82.74 |
| B (MnP) | -*- | 98.82 (97.67,99.97) | -*- | 99.70 (99.12,100.00) | 0.29 (-0.28,0.87) | 49.41 |
| B (MA) | 100.00 (100.00,100.00) | 97.51 (95.38,99.65) | 37.50 (30.86,44.14) | 100.00 (100.00,100.00) | 1.47 (-0.18,3.12) | 98.76 |
| B (MnA) | -*- | -*- | -*- | -*- | -*- | -*- |
| C (MP) | 28.89 (24.07,33.71) | 95.93 (93.83,98.03) | 52.00 (46.69,57.31) | 89.84 (86.63,93.05) | 13.24 (9.63,16.84) | 62.41 |
| C (MnP) | 36.73 (31.61,41.86) | 94.50 (92.08,96.92) | 52.94 (47.64,58.25) | 89.87 (86.66,93.08) | 14.41 (10.68,18.14) | 65.62 |
| C (MA) | 30.77 (24.44,37.10) | 97.38 (95.19,99.57) | 44.44 (37.63,51.26) | 95.38 (92.51,98.26) | 6.37 (3.02,9.72) | 72.77 |
| C (MnA) | 50.00 (43.14,56.86) | 95.54 (92.71,98.38) | 10.00 (5.88,14.12) | 99.48 (98.50,100.00) | 0.98 (-0.37,2.33) | 72.77 |
| K (MP) | 36.51 (31.39,41.63) | 93.14 (90.45,95.83) | 54.76 (49.47,60.05) | 86.58 (82.95,90.20) | 18.53 (14.40,22.66) | 64.82 |
| K (MnP) | 24.24 (19.69,28.80) | 95.62 (93.45,97.80) | 57.14 (51.88,62.40) | 83.97 (80.08,87.87) | 19.41 (15.21,23.62) | 59.93 |
| K (MA) | 24.32 (18.44,30.21) | 91.02 (87.09,94.94) | 37.50 (30.86,44.14) | 84.44 (79.47,89.42) | 18.14 (12.85,23.42) | 57.67 |
| K (MnA) | 48.39 (41.53,55.24) | 85.59 (80.77,90.41) | 73.77 (67.73,79.81) | 66.43 (59.95,72.91) | 45.59 (38.75,52.42) | 66.99 |
| P (MP) | 31.71 (26.76,36.65) | 76.25 (71.73,80.78) | 15.48 (11.63,19.32) | 89.06 (85.74,92.38) | 12.06 (8.60,15.52) | 53.98 |
| P (MnP) | 67.74 (62.77,72.71) | 72.82 (68.09,77.54) | 20.00 (15.75,24.25) | 95.74 (93.60,97.89) | 9.12 (6.06,12.18) | 70.28 |
| P (MA) | -*- | 96.04 (93.39,98.73) | -*- | 99.49 (98.51,100.00) | 0.49 (-0.47, 1.45) | 48.03 |
| P (MnA) | -*- | 98.53 (-*-) | -*- | 99.49 (98.50,100.47) | -*- | -*- |
| R (MP) | 57.14 (51.88,62.40) | 80.56 (76.36,84.77) | 16.22 (12.30,20.13) | 96.62 (94.69,98.54) | 6.18 (3.62,8.74) | 68.85 |
| R (MnP) | 58.33 (53.09,63.57) | 81.01 (76.84,85.18) | 18.92 (14.76,23.08) | 96.24 (94.22,98.26) | 7.06 (4.34,9.78) | 69.67 |
| R (MA) | 67.57 (61.14,73.99) | 55.09 (48.26,61.92) | 25.00 (19.06,30.94) | 88.46 (84.08,92.85) | 18.14 (12.85, 23.42) | 61.33 |
| R (MnA) | 58.97 (52.22,65.72) | 62.70 (56.06,69.33) | 49.46 (42.60,56.32) | 71.17 (64.96,77.39) | 38.24 (31.57,44.90) | 60.84 |
| S (MP) | 13.42 (9.80,17.05) | 98.95 (97.87,100.00) | 90.91 (87.85,93.96) | 59.43 (54.21,64.65) | 43.82 (38.55,49.10) | 56.19 |
| S (MnP) | 12.75 (9.21,16.30) | 99.48 (98.71,100.00) | 95.00 (92.68,97.32) | 59.38 (54.15,64.60) | 43.82 (38.55,49.10) | 56.11 |
| S (MA) | 42.64 (35.85,49.42) | 97.33 (95.12,99.54) | 96.49 (93.97,99.02) | 49.66 (42.80,56.52) | 63.24 (56.62,69.85) | 69.98 |
| S (MnA) | 35.11 (28.56,41.66) | 98.18 (96.35,100.00) | 94.29 (91.10,97.47) | 63.91 (57.31,70.50) | 46.08 (39.24,52.92) | 66.64 |
| X (MP) | 100.00 (100.00,100.00) | 99.40 (98.58,100.00) | 71.43 (66.63,76.23) | 100.00 (100.00,100.00) | 1.47 (0.19,2.75) | 99.70 |
| X (MnP) | 50.00 (44.69,55.31) | 98.58 (97.21,99.79) | 37.50 (32.35,42.65) | 99.10 (98.09,100.00) | 1.76 (0.37,3.16) | 74.25 |
| X (MA) | -*- | 99.51 (-*-) | -*- | -*- | -*- | -*- |
| X (MnA) | -*- | 91.18 (-*-) | -*- | -*- | -*- | -*- |

*B = Bleeding; C = Caries/Secondary caries; K = Calculus; P = Pit and fissure caries/stain; R = Gingival recession; S = Staining; X = Retained root; MP = Maxillary posterior; MnP = Mandibular posterior; MA = Maxillary anterior; MnA = Mandibular anterior; O = Overall. *Not enough datapoint for calculation of the outcome in this region*

Supplementary Table 2: Overall and Region-wise results for Subgroup Analysis with Prevalence and AUC values.

| **Outcome** | **Sensitivity %** | **Specificity %** | **PPV %** | **NPV %** | **Prevalence %** | **AUC Values** |
| --- | --- | --- | --- | --- | --- | --- |
| **B (MP)** | 66.67  (61.06, 72.27) | 98.51  (97.07, 99.95) | 33.33  (27.73, 38.94) | 99.62  (98.90, 100.00) | 1.10 (-0.14,2.34) | 82.74 |
| **B (MnP)** | 0.00  (0.00, 0.00) | 98.46  (96.96, 99.95) | 0.00  (0.00, 0.00) | 99.61  (98.85, 100.00) | 0.38 (-0.37,1.14) | 49.41 |
| **B (MA)** | 100.00  (100.00, 100.00) | 97.50  (95.35, 99.65) | 37.50  (30.84, 44.16) | 100.00  (100.00, 100.00) | 1.48 (-0.18,3.14) | 98.76 |
| **B (MnA)** | -*- | -*- | -*- | -*- | -*- | -*- |
| **B (O)** | 71.43  (68.54, 74.32) | 98.60  (97.85, 99.35) | 27.78  (24.91, 30.65) | 99.78  (99.48, 100.00) | 0.75 (0.20,1.30) | 85.11 |
| **C (MP)** | 28.89  (23.50, 34.28) | 94.71  (92.05, 97.37) | 52.00  (46.06, 57.94) | 87.04  (83.05, 91.04) | 16.54 (12.13,20.96) | 62.41 |
| **C (MnP)** | 36.73  (30.87, 42.59) | 92.42  (89.20, 95.63) | 52.94  (46.87, 59.01) | 86.28  (82.10, 90.46) | 18.85 (14.09,23.60) | 65.62 |
| **C (MA)** | 30.77  (24.42, 37.12) | 97.37  (95.17, 99.57) | 44.44  (37.61, 51.28) | 95.36  (92.47, 98.25) | 6.40 (3.04,9.77) | 64.08 |
| **C (MnA)** | 50.00  (43.10, 56.90) | 95.04  (92.64, 98.36) | 10.00  (5.86, 14.14) | 99.48  (98.49, 100.47) | 0.99 (-0.38,2.36) | 72.77 |
| **C (O)** | 33.03  (30.02, 36.04) | 94.93  (93.52, 96.33) | 46.15  (42.96, 49.35) | 91.50  (89.72, 93.29) | 11.63 (9.58,13.69) | 64.37 |
| **K (MP)** | 36.51  (30.79, 42.23) | 90.91  (87.49, 94.33) | 54.76  (48.85, 60.68) | 82.61  (78.10, 87.11) | 23.16 (18.15,28.18) | 64.82 |
| **K (MnP)** | 24.24  (19.03, 29.45) | 93.81  (90.89, 96.74) | 57.14  (51.13, 63.16) | 78.45  (73.45, 83.45) | 25.38 (20.09,30.67) | 59.93 |
| **K (MA)** | 24.32  (18.42, 30.23) | 90.96  (87.02, 94.91) | 37.50  (30.84, 44.16) | 84.36  (79.36, 89.35) | 18.23 (12.92,23.54) | 57.67 |
| **K (MnA)** | 48.39  (41.50, 55.28) | 85.32  (80.44, 90.20) | 73.77  (67.70, 79.84) | 65.96  (59.42, 72.49) | 46.04 (39.17,52.91) | 66.99 |
| **K (O)** | 35.91  (32.84, 38.98) | 90.86  (89.01, 92.70) | 60.00  (56.86, 63.14) | 78.77  (76.15, 81.39) | 27.64 (24.78,30.50) | 64.21 |
| **P (MP)** | 31.71  (26.18, 37.24) | 69.26  (63.78, 74.75) | 15.48  (11.18, 19.77) | 85.11  (80.88, 89.34) | 15.07 (10.82,19.33) | 53.98 |
| **P (MnP)** | 67.74  (62.06, 73.42) | 63.32  (57.46, 69.18) | 20.00  (15.14, 24.86) | 93.55  (90.56, 96.53) | 11.92 (7.98,15.86) | 70.28 |
| **P (MA)** | 0.00  (0.00, 0.00) | 96.04  (93.36, 98.72) | 0.00  (0.00, 0.00) | 99.49  (98.50, 100.00) | 0.49 (-0.47,1.46) | 48.03 |
| **P (MnA)** | 0.00  (0.00, 0.00) | 96.04  (93.36, 98.72) | 0.00  (0.00, 0.00) | 99.49  (98.50, 100.00) | 0.49 (-0.47,1.46) | -*- |
| **P (O)** | 45.95  (42.76, 49.14) | 80.21  (77.66, 82.76) | 16.59  (14.21, 18.97) | 94.54  (93.09, 96.00) | 7.89 (6.16,9.61) | 65.11 |
| **R (MP)** | 57.14  (51.26, 63.02) | 75.30  (70.17, 80.42) | 16.22  (11.84, 20.60) | 95.45  (92.98, 97.93) | 7.72 (4.55,10.89) | 68.85 |
| **R (MnP)** | 58.33  (52.34, 64.33) | 74.58  (69.28, 79.87) | 18.92  (14.16, 23.68) | 94.62  (91.88, 97.37) | 9.23 (5.71,12.75) | 69.67 |
| **R (MA)** | 67.57  (61.13, 74.01) | 54.82  (47.97, 61.67) | 25.00  (19.04, 30.96) | 88.35  (83.94, 92.76) | 18.23 (12.92,23.54) | 61.33 |
| **R (MnA)** | 60.00  (52.59, 67.41) | 59.22  (51.79, 66.65) | 48.15  (40.59, 55.70) | 70.11  (63.19, 77.04) | 38.69 (31.33,46.06) | 60.84 |
| **R (O)** | 61.22  (58.05, 64.40) | 68.39  (65.35, 71.42) | 27.36  (24.45, 30.26) | 90.07  (88.12, 92.02) | 16.28 (13.87,18.69) | 67.17 |
| **S (MP)** | 13.42  (9.37, 17.47) | 98.37  (96.87, 99.88) | 90.91  (87.49, 94.33) | 48.40  (42.46, 54.34) | 54.78 (48.86,60.69) | 56.19 |
| **S (MnP)** | 12.75  (8.70, 16.81) | 99.10  (97.95, 100.00) | 95.00  (92.35, 97.65) | 45.83  (39.78, 51.89) | 57.31 (51.30,63.32) | 56.11 |
| **S (MA)** | 42.64  (35.83, 49.44) | 97.30  (95.07, 99.53) | 96.49  (93.96, 99.02) | 49.32  (42.44, 56.19) | 63.55 (56.93,70.17) | 69.98 |
| **S (MnA)** | 35.11  (28.52, 41.69) | 98.15  (96.29, 100.00) | 94.29  (91.08, 97.49) | 63.47  (56.83, 70.11) | 46.53 (39.66,53.41) | 66.64 |
| **S (O)** | 24.38  (21.63, 27.13) | 98.32  (97.49, 99.14) | 94.78  (93.35, 96.20) | 50.93  (47.73, 54.13) | 55.60 (52.42,58.78) | 61.57 |
| **X (MP)** | 100.00  (100.00, 100.00) | 99.25  (98.23, 100.00) | 71.43  (66.06, 76.80) | 100.00  (100.00, 100.00) | 1.84 (0.24,3.43) | 99.70 |
| **X (MnP)** | 50.00  (43.92, 56.08) | 98.03  (96.34, 99.72) | 37.50  (31.62, 43.38) | 98.81  (97.49, 100.00) | 2.31 (0.48,4.13) | 74.25 |
| **X (MA)** | 99.51 | -*- | -*- | -*- | -*- | -*- |
| **X (MnA)** | 91.09 | -*- | -*- | -*- | -*- | -*- |
| **X (O)** | 72.73  (68.88, 75.58) | 97.19  (96.13, 98.25) | 23.53  (20.81, 26.25) | 99.67  (99.30, 100.00) | 1.17 (0.48,1.86) | 85.16 |

*B = Bleeding; C = Caries/Secondary caries; K = Calculus; P = Pit and fissure caries/stain; R = Gingival recession; S = Staining; X = Retained root; MP = Maxillary posterior; MnP = Mandibular posterior; MA = Maxillary anterior; MnA = Mandibular anterior; O = Overall. *Not enough datapoint for calculation of the outcome in this region*

## Supplementary Figures

**Main group analysis ROC curves and AUC values:**


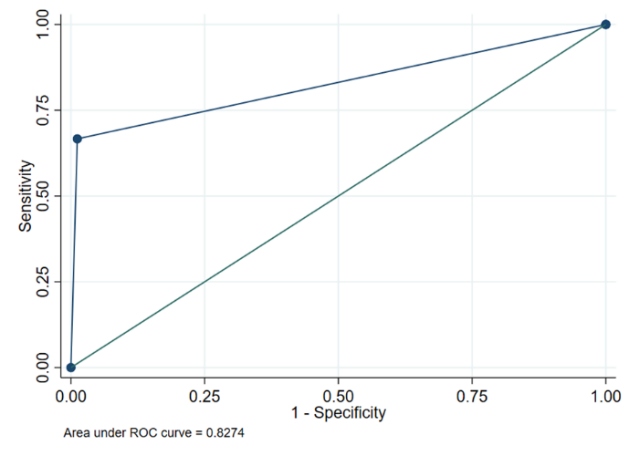


Figure 6: Bleeding ROC curve for maxillary posterior region.


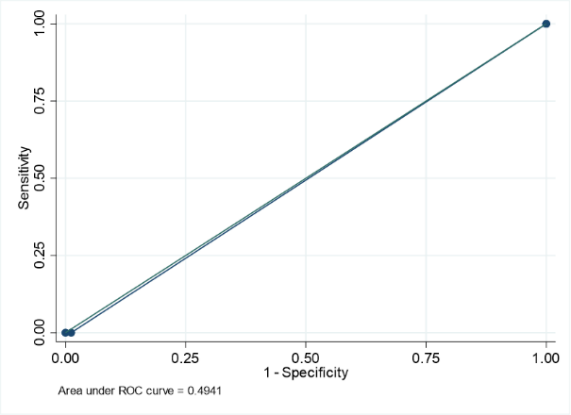


Figure 7: Bleeding ROC curve for mandibular posterior region.


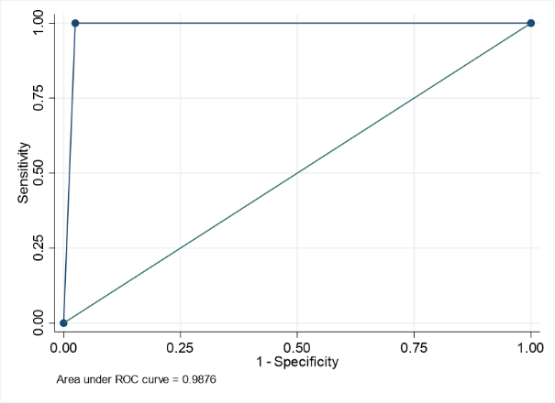


Figure 8: Bleeding ROC curve for maxillary anterior region.


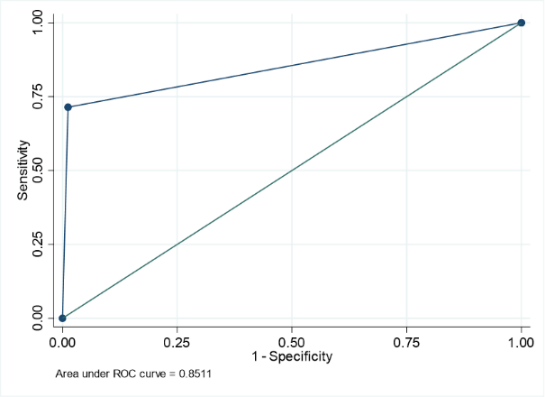


Figure 9: Overall Bleeding ROC curve.


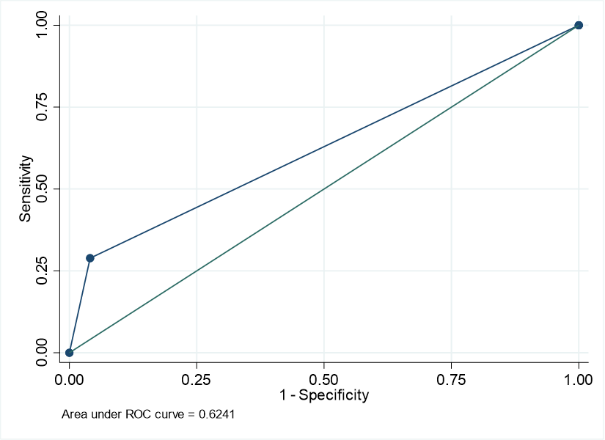


Figure 10: Caries ROC curve for maxillary posterior region.


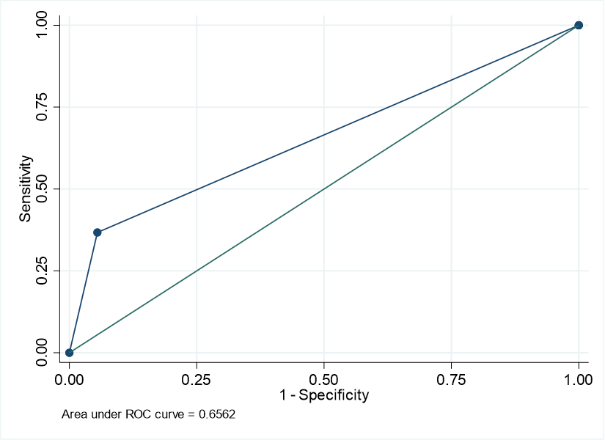


Figure 11: Caries ROC curve for mandibular posterior region.

Figure 12: Caries ROC curve for maxillary anterior region.


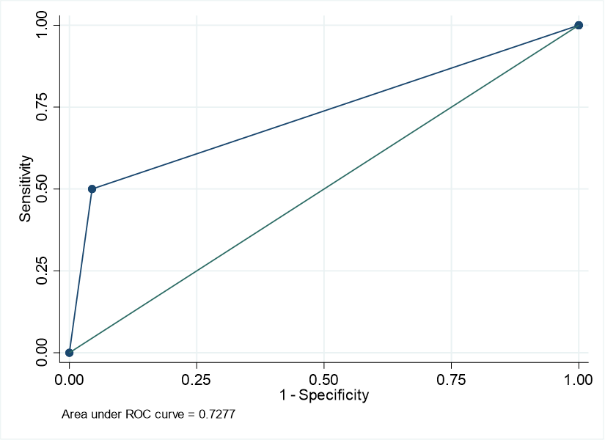


Figure 13: Caries ROC curve for mandibular anterior region.


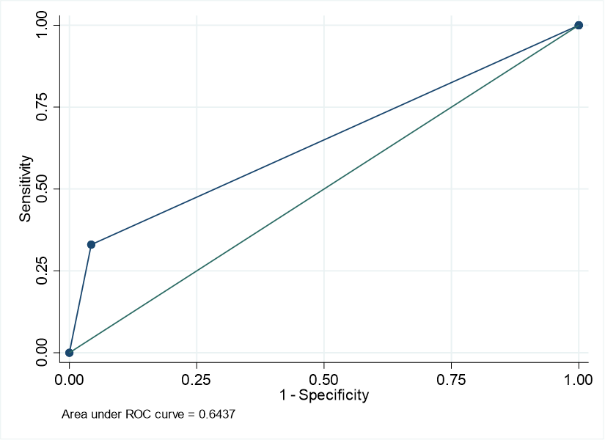


Figure 14: Overall Caries ROC curve.


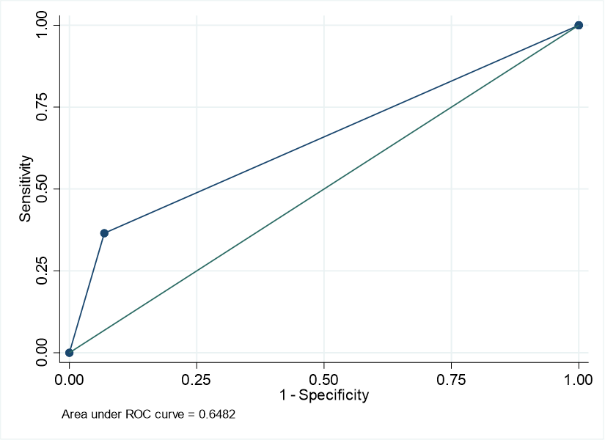


Figure 15: Calculus ROC curve for maxillary posterior region.


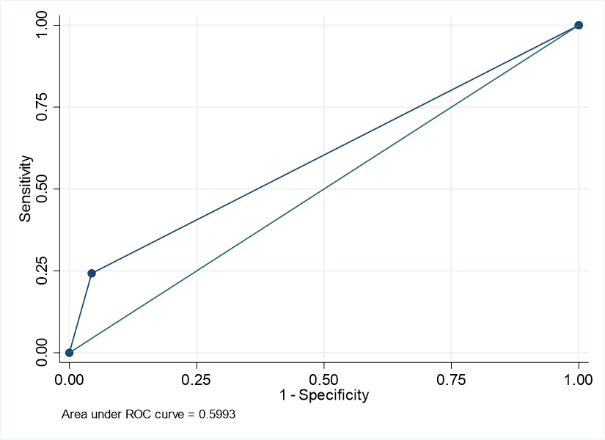


Figure 16: Calculus ROC curve for mandibular posterior region.


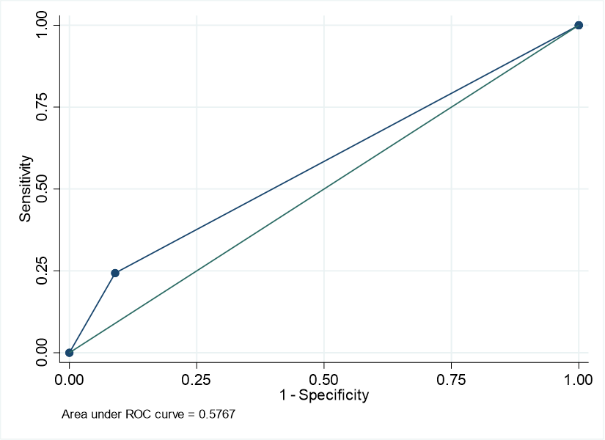


Figure 17: Calculus ROC curve for maxillary anterior region.


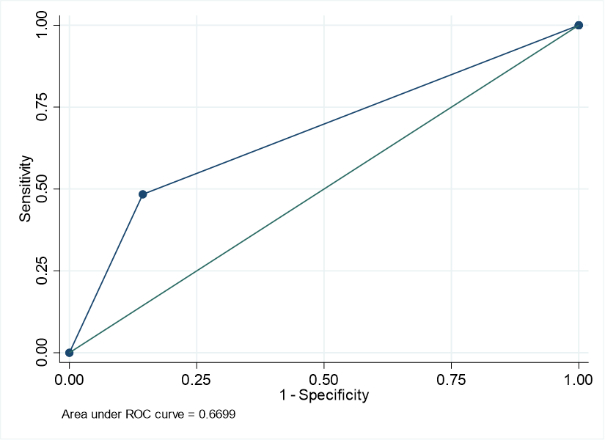


Figure 18: Calculus ROC curve for mandibular anterior region.


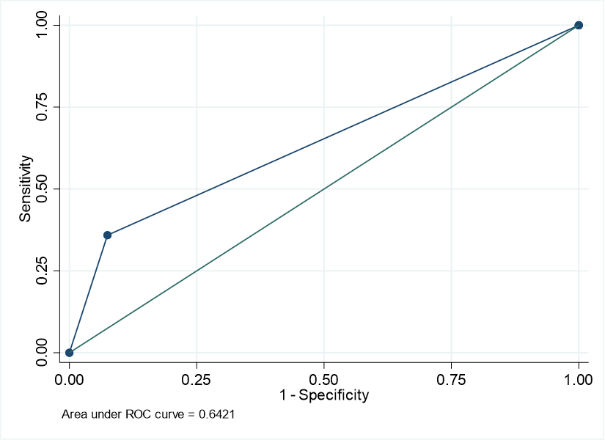


Figure 19: Overall Calculus ROC curve.


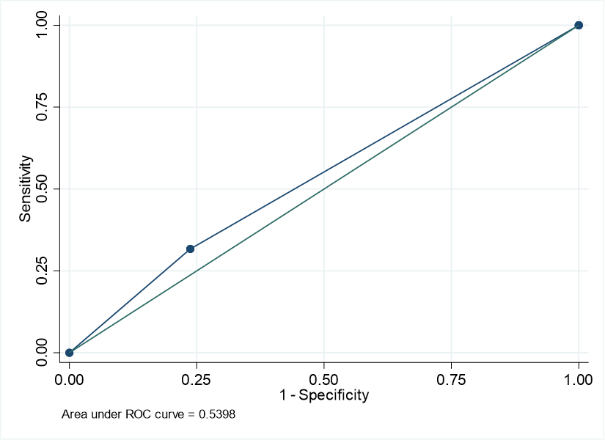


Figure 20: Pit and fissure/Stain ROC curve for maxillary posterior region.


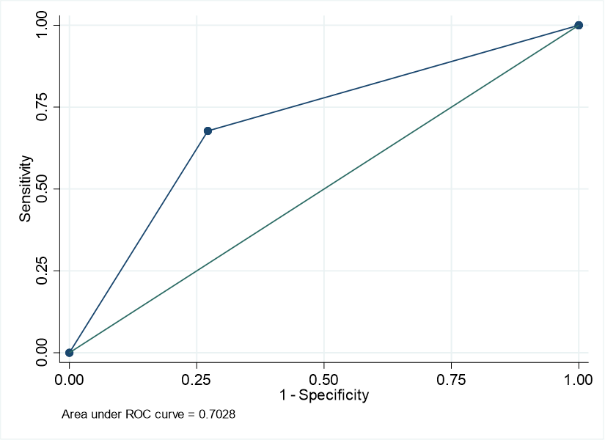


Figure 21: Pit and fissure/Stain ROC curve for mandibular posterior region.


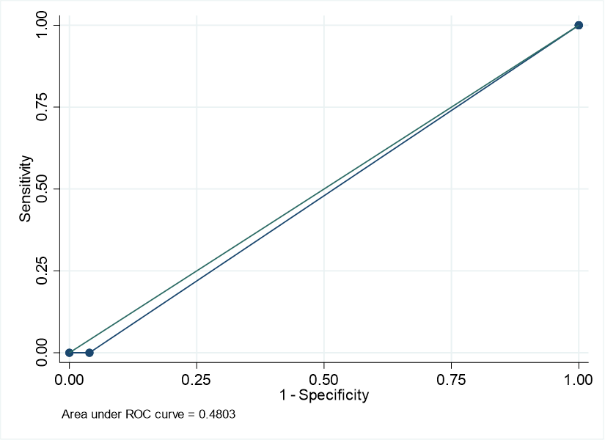


Figure 22: Pit and fissure/Stain ROC curve for maxillary anterior region.


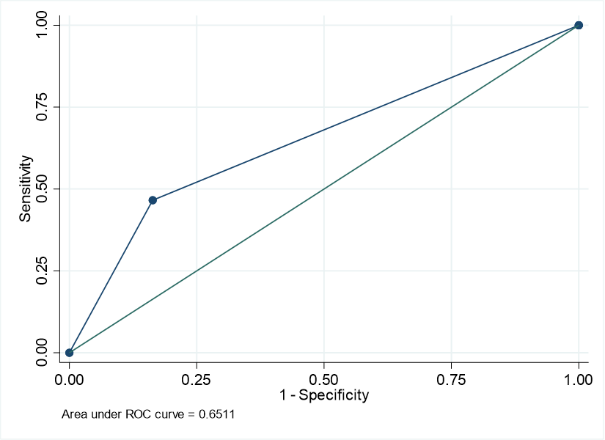


Figure 23: Overall Pit and fissure/Stain ROC curve.


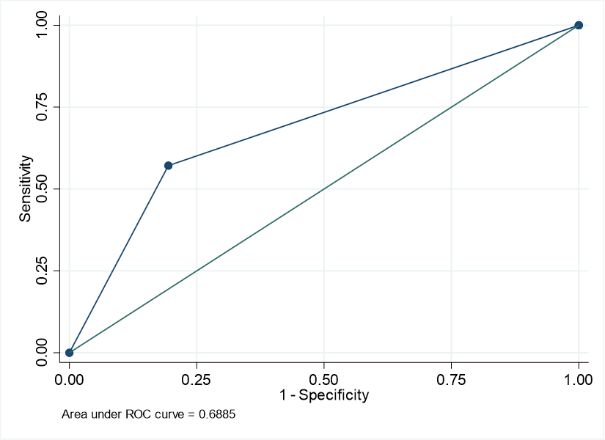


Figure 24: Recession ROC curve for maxillary posterior region.


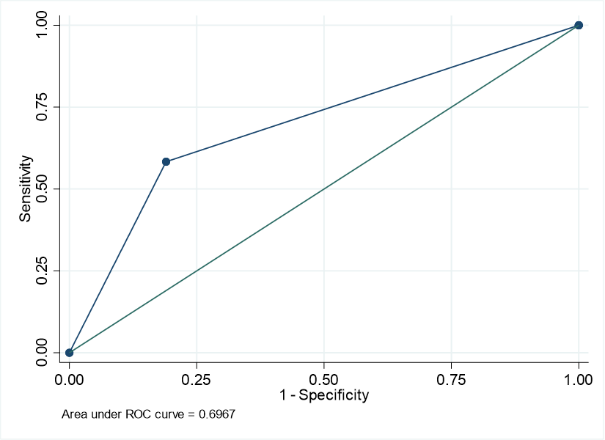


Figure 25: Recession ROC curve for mandibular posterior region.


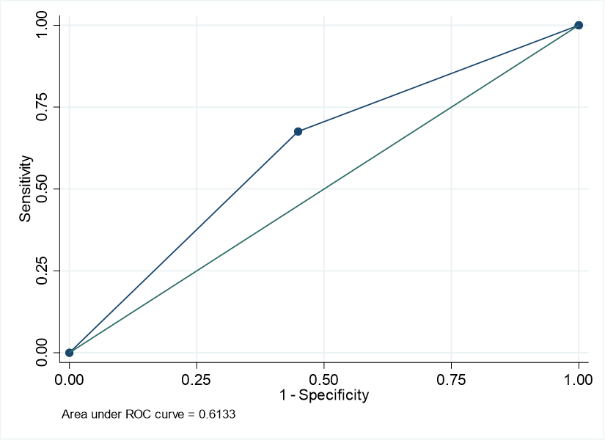


Figure 26: Recession ROC curve for maxillary anterior region.


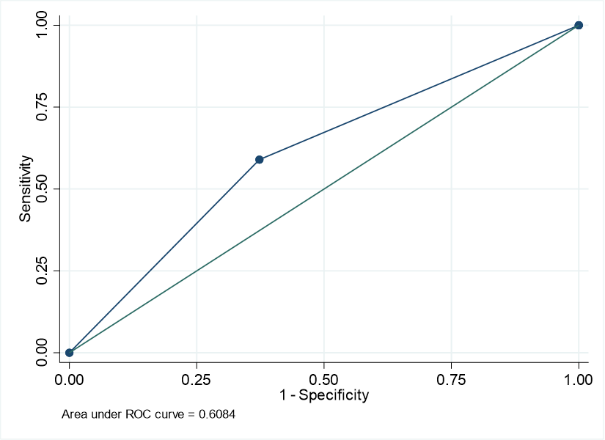


Figure 27: Recession ROC curve for mandibular anterior region.


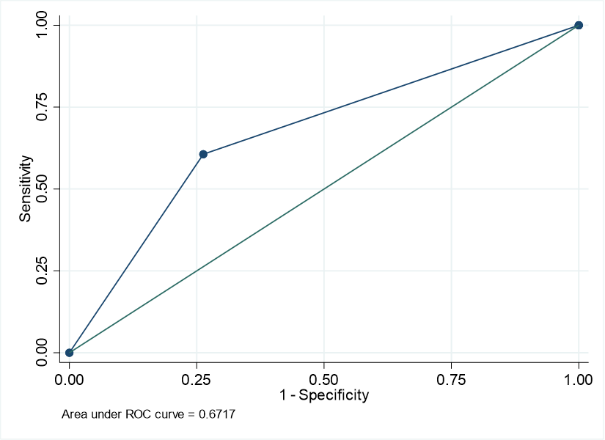


Figure 28: Overall Recession ROC curve.


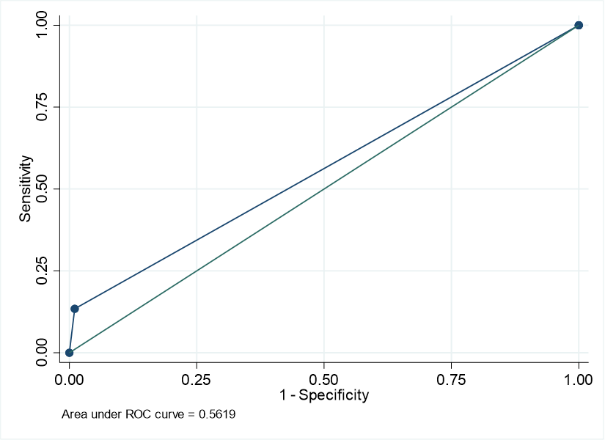


Figure 29: Smooth surface stain ROC curve for maxillary posterior region.


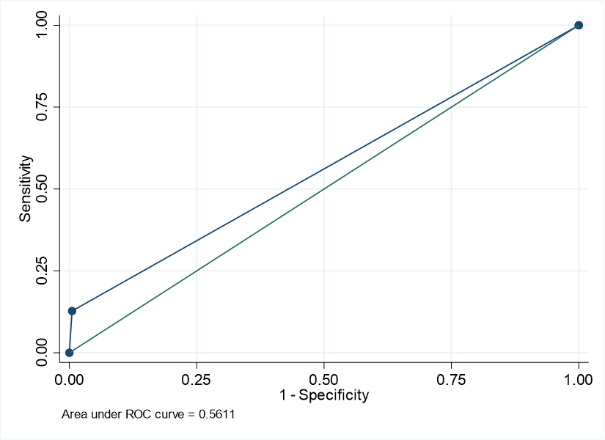


Figure 30: Smooth surface stain ROC curve for mandibular posterior region.


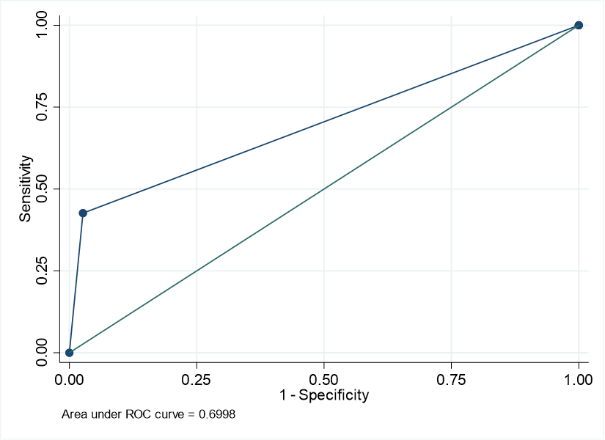


Figure 31: Smooth surface stain ROC curve for maxillary anterior region.


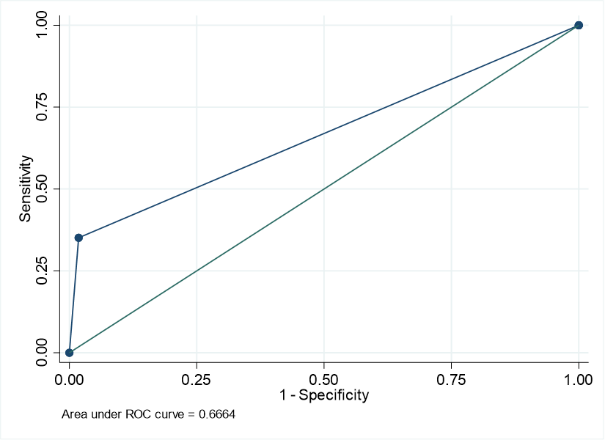


Figure 32: Smooth surface stain ROC curve for mandibular anterior region.


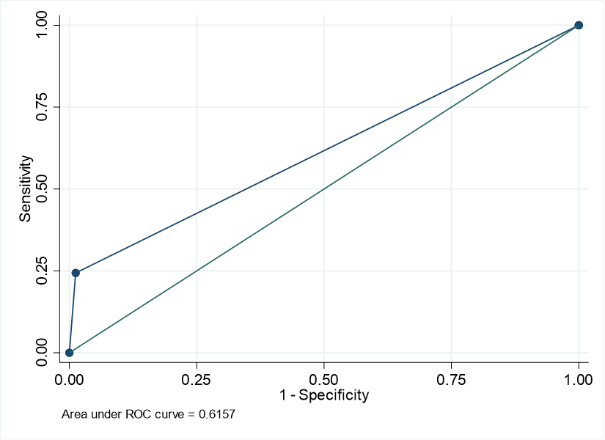


Figure 33: Overall Smooth surface stain ROC curve.


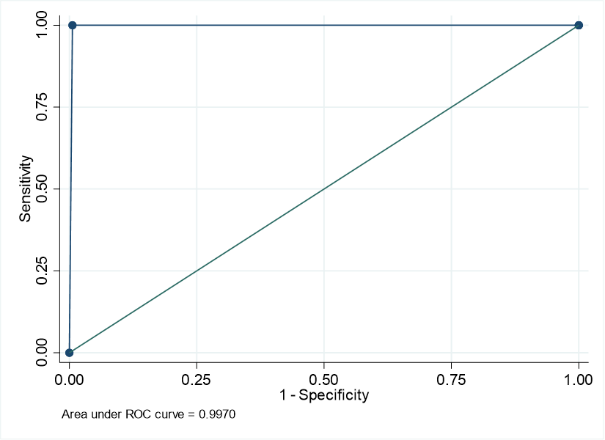


Figure 34: Retained root ROC curve for maxillary posterior region.


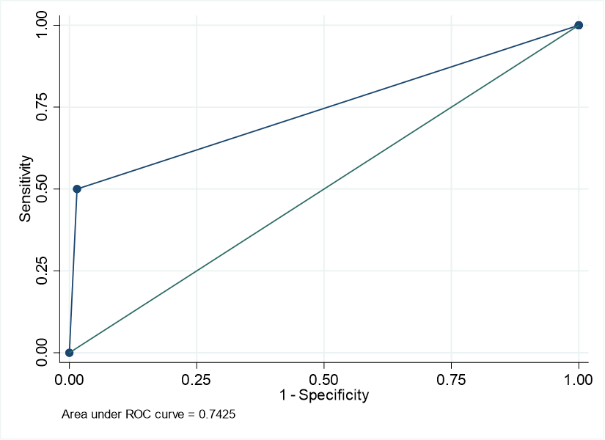


Figure 35: Retained root ROC curve for mandibular posterior region.


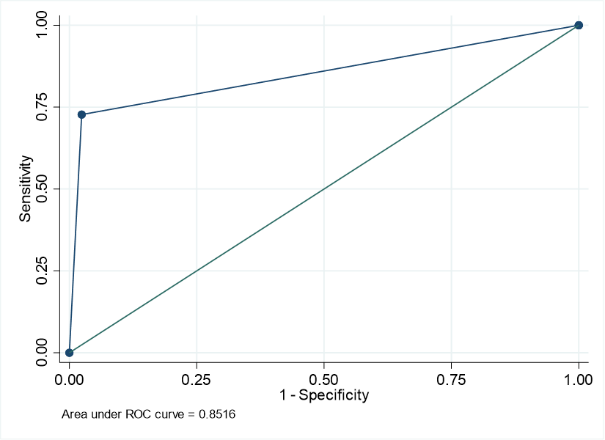


Figure 36: Overall Retained root ROC curve.

**Subgroup analysis ROC curves AUC values:**

Figure 37: Bleeding ROC curve for maxillary posterior region.


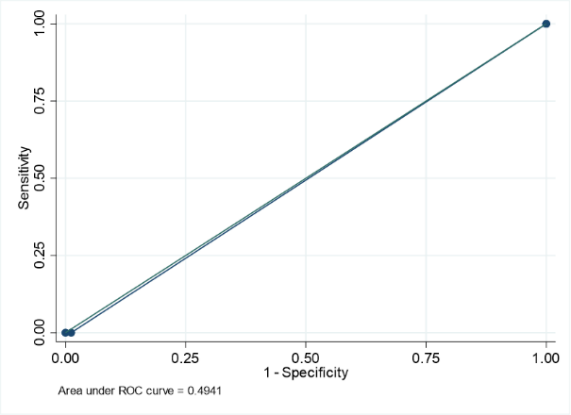


Figure 38: Bleeding ROC curve for mandibular posterior region.


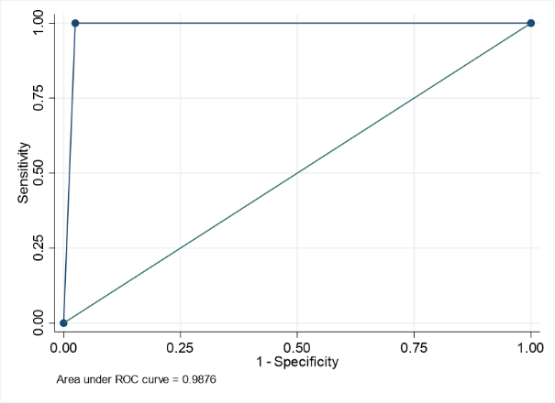


Figure 39: Bleeding ROC curve for maxillary anterior region.


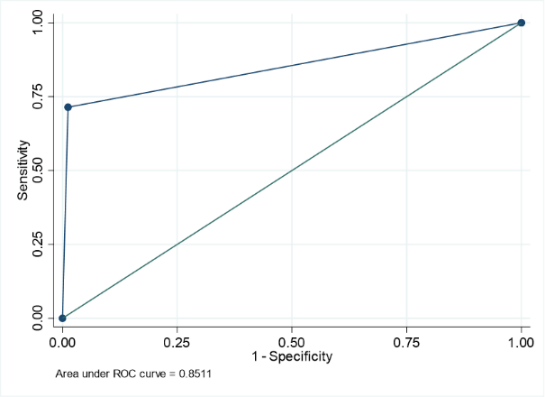


Figure 40: Overall Bleeding ROC curve.


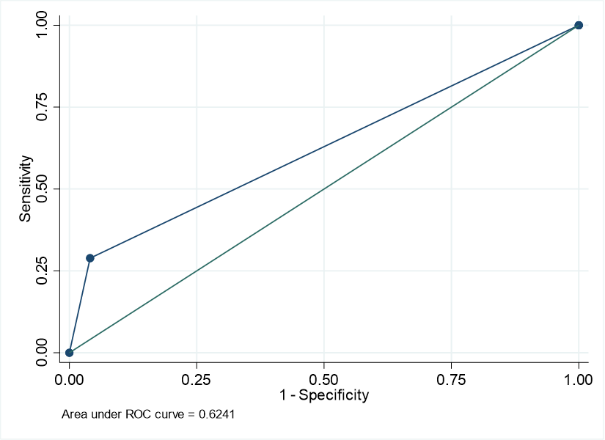


Figure 41: Caries ROC curve for maxillary posterior region.


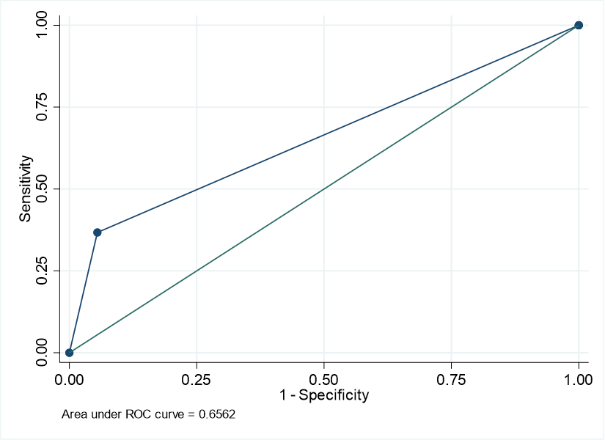


Figure 42: Caries ROC curve for mandibular posterior region.

Figure 43: Caries ROC curve for maxillary anterior region.


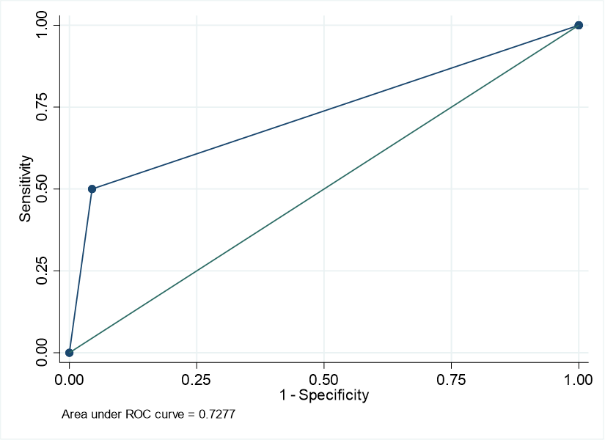


Figure 44: Caries ROC curve for mandibular anterior region.


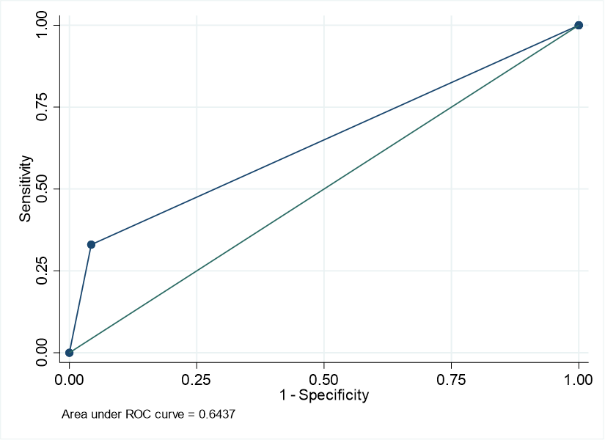


Figure 45: Overall Caries ROC curve.


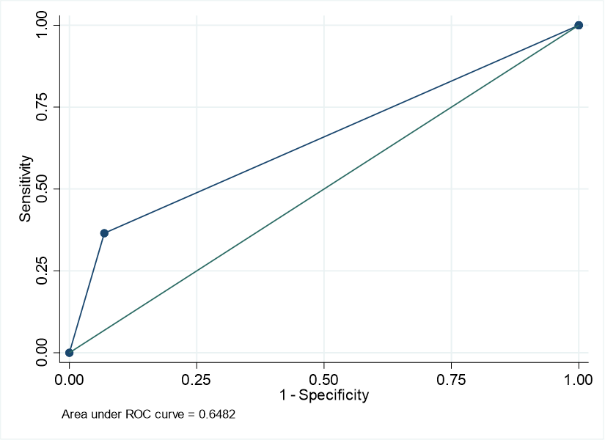


Figure 46: Calculus ROC curve for maxillary posterior region.


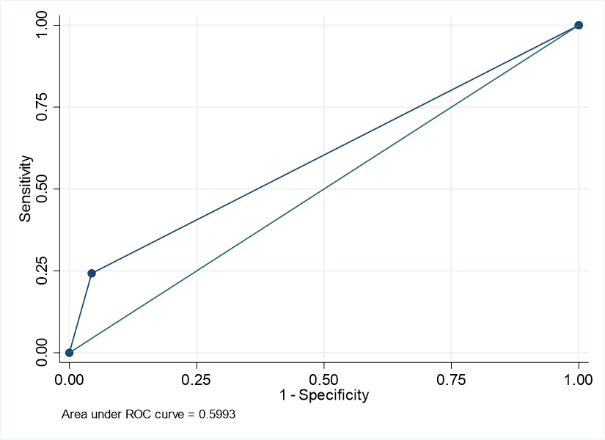


Figure 47: Calculus ROC curve for mandibular posterior region.


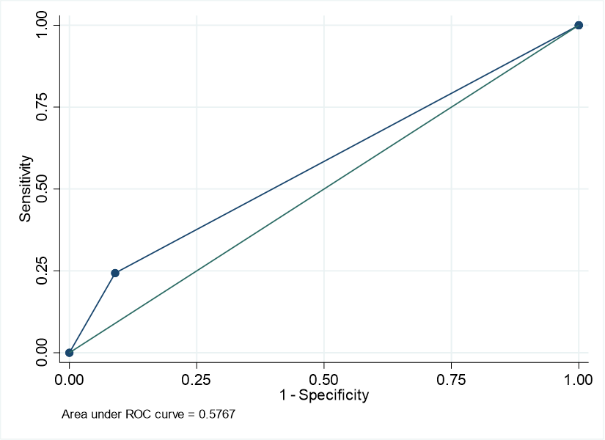


Figure 48: Calculus ROC curve for maxillary anterior region.


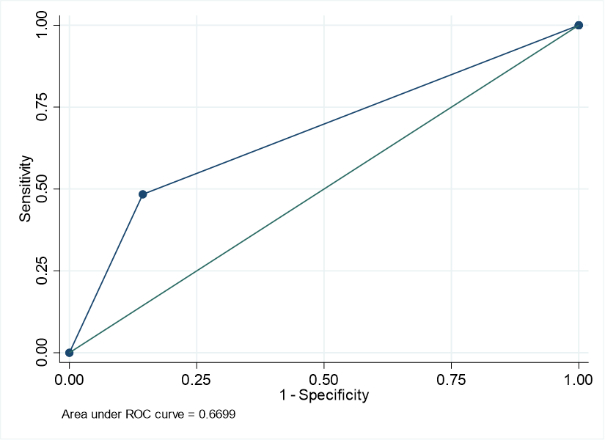


Figure 49: Calculus ROC curve for mandibular anterior region.


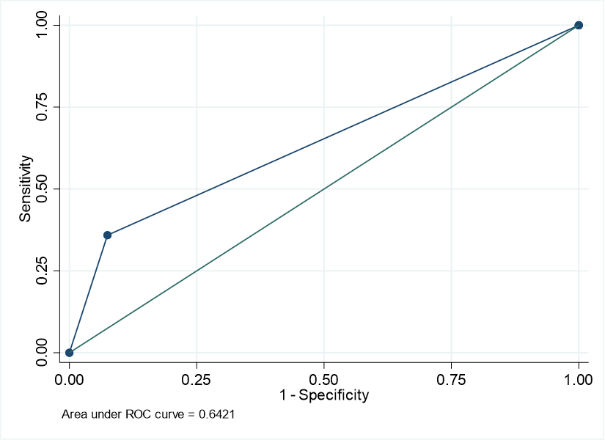


Figure 50: Overall Calculus ROC curve.


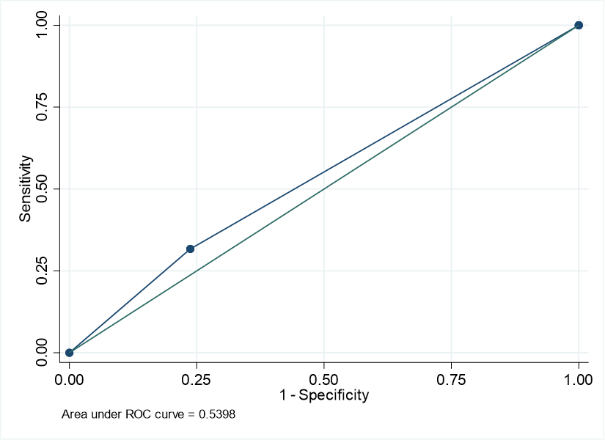


Figure 51: Pit and fissure/Stain ROC curve for maxillary posterior region.


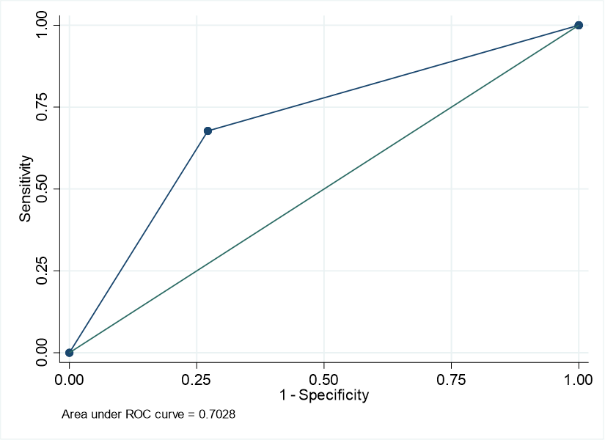


Figure 52: Pit and fissure/Stain ROC curve for mandibular posterior region.


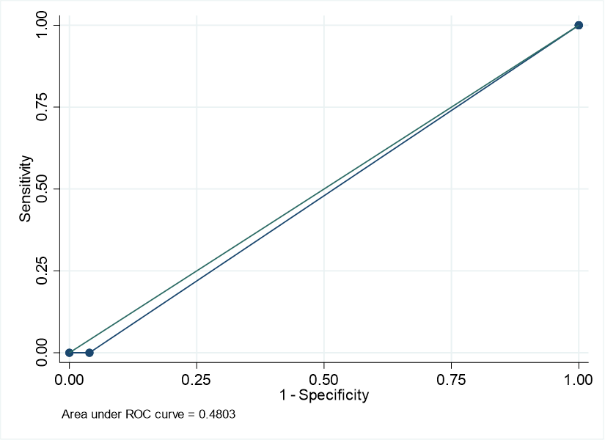


Figure 53: Pit and fissure/Stain ROC curve for maxillary anterior region.

Figure 54: Pit and fissure/Stain ROC curve for mandibular anterior region.


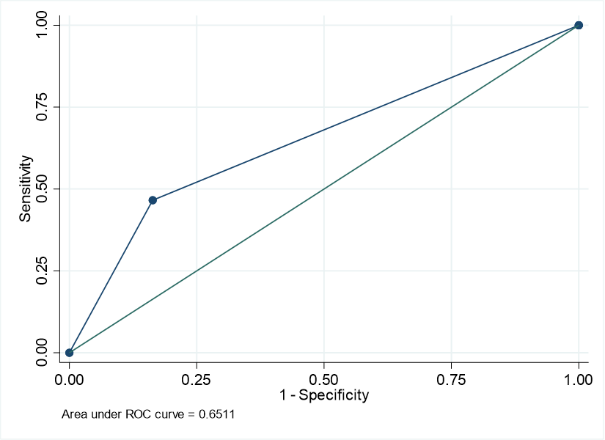


Figure 55: Overall Pit and fissure/Stain ROC curve.


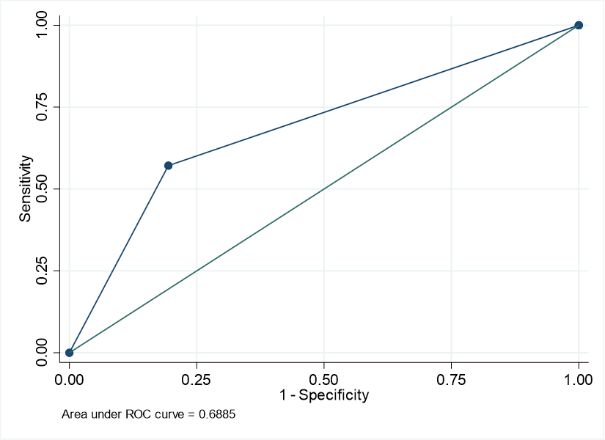


Figure 56: Recession ROC curve for maxillary posterior region.


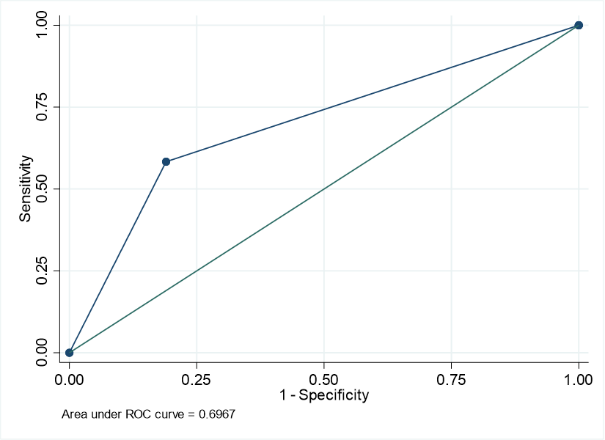


Figure 57: Recession ROC curve for mandibular posterior region.


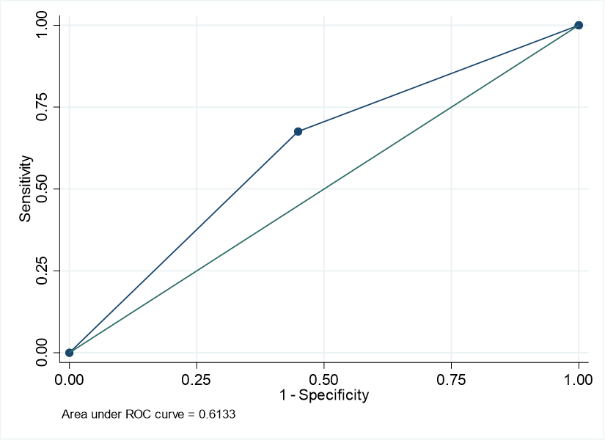


Figure 58: Recession ROC curve for maxillary anterior region.


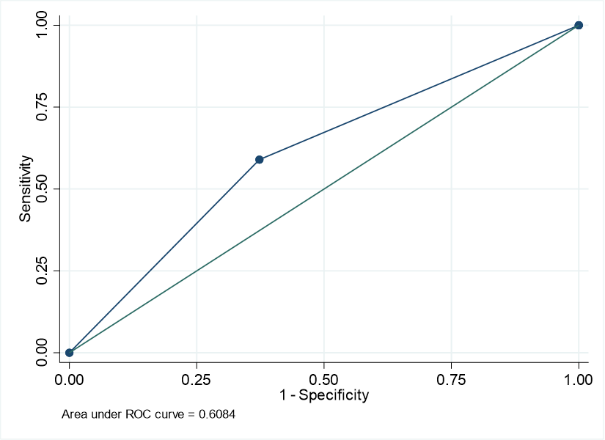


Figure 59: Recession ROC curve for mandibular anterior region.


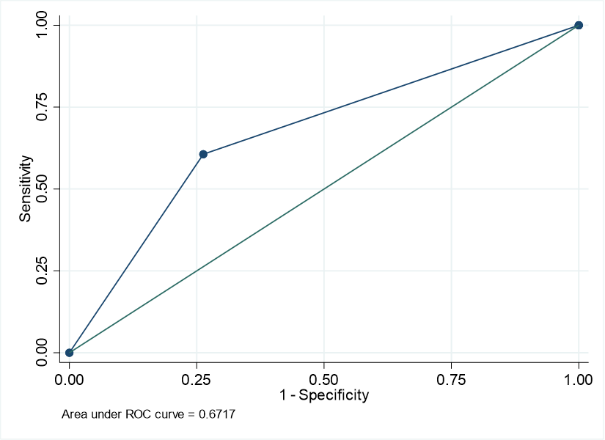


Figure 60: Overall Recession ROC curve.


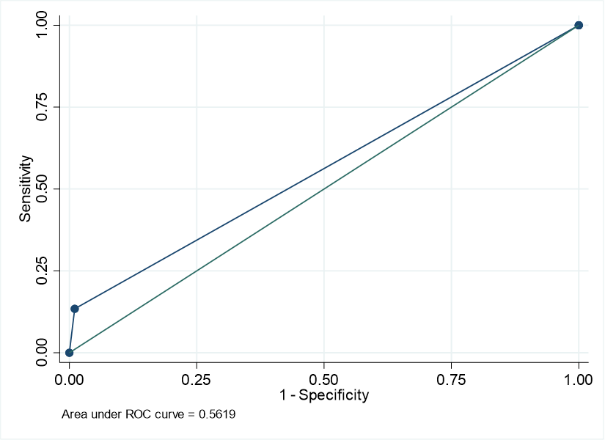


Figure 61: Smooth surface stain ROC curve for maxillary posterior region.


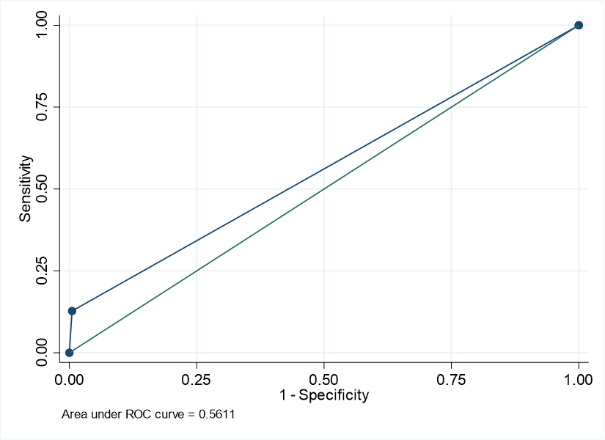


Figure 62: Smooth surface stain ROC curve for mandibular posterior region.


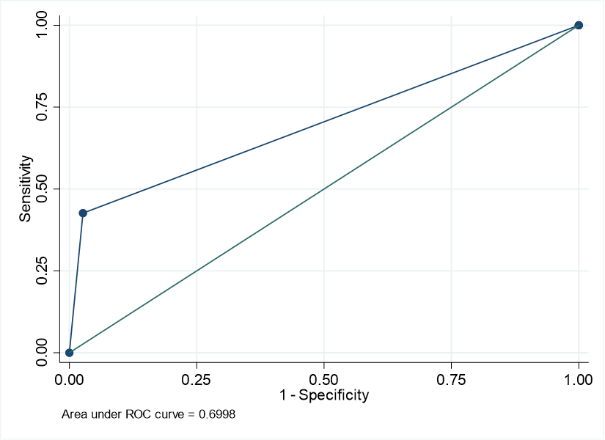


Figure 63: Smooth surface stain ROC curve for maxillary anterior region.


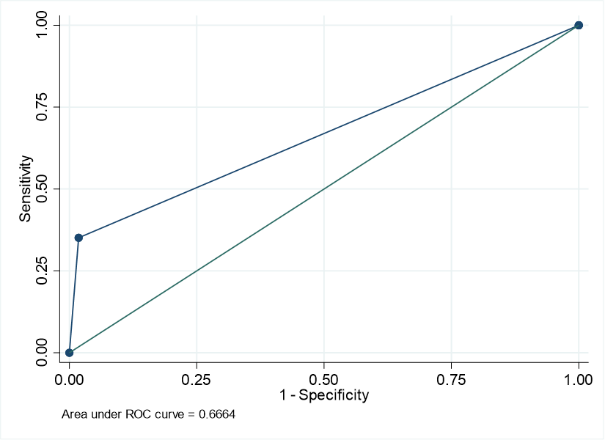


Figure 64: Smooth surface stain ROC curve for mandibular anterior region.


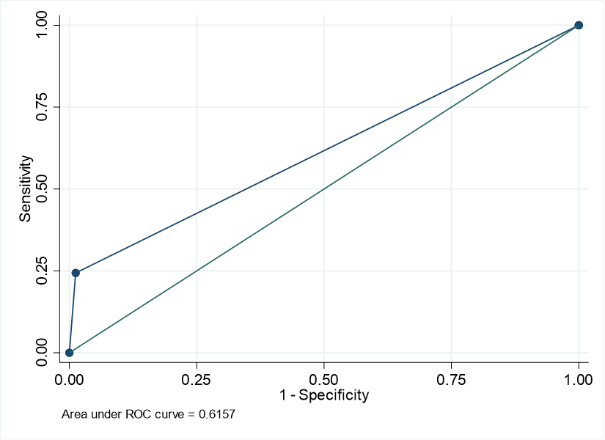


Figure 65: Overall Smooth surface stain ROC curve.


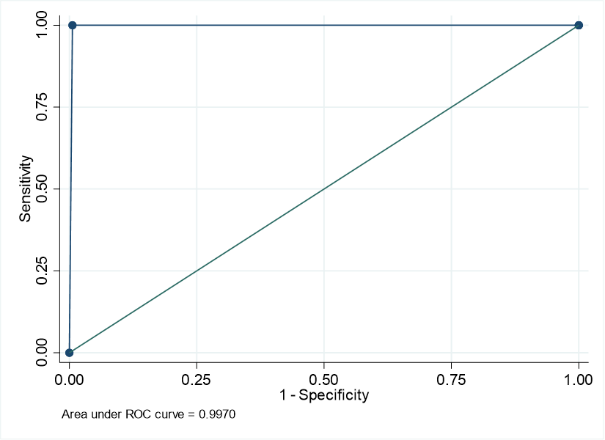


Figure 66: Retained root ROC curve for maxillary posterior region.


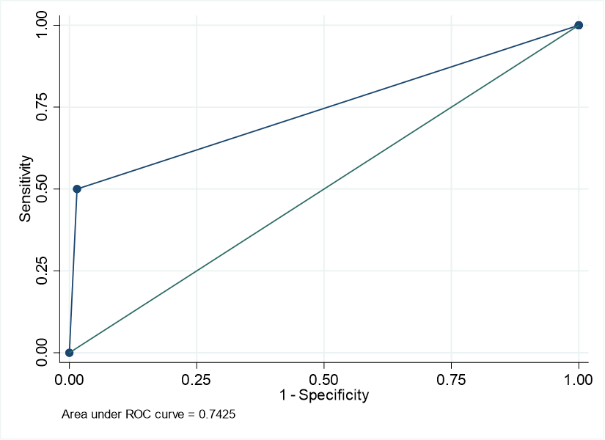


Figure 67: Retained root ROC curve for mandibular posterior region.


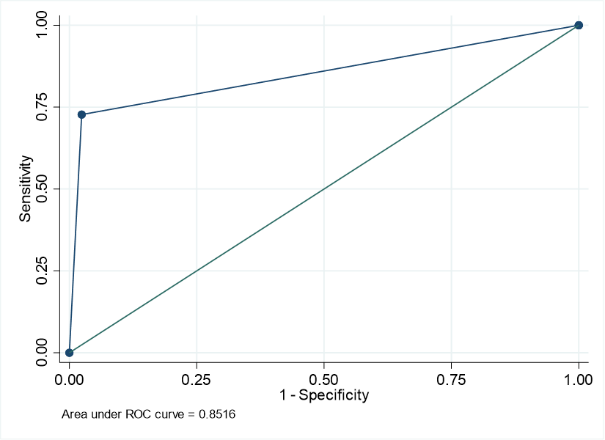


Figure 68: Overall Retained root ROC curve.
